# Upper gastrointestinal endoscopic screening uptake and associated factors in East Asia: a systematic review and meta-analysis

**DOI:** 10.3389/fpubh.2026.1869526

**Published:** 2026-07-29

**Authors:** Yanjie Liu, Yan He, Yuan Zhao, Wenhao Tian, Wanya Pan, Yunyu Guo, Meiling Xie, Jing Hua, Xiuqin Feng

**Affiliations:** 1 Department of Nursing, The Second Affiliated Hospital Zhejiang University School of Medicine (SAHZU), Hangzhou, Zhejiang, China; 2 School of Nursing, Zhejiang University School of Medicine, Zhejiang University, Hangzhou, Zhejiang, China

**Keywords:** endoscopic uptake, influencing factors, meta-analysis, systematic review, upper gastrointestinal cancer screening

## Abstract

**Objective:**

To estimate the pooled uptake of upper gastrointestinal endoscopic screening uptake and to identify factors associated with screening uptake among populations eligible for upper gastrointestinal endoscopic screening.

**Methods:**

A systematic review and meta-analysis was conducted to identify studies reporting the uptake of upper gastrointestinal endoscopic screening and factors associated with screening participation. The pooled uptake was calculated using the random-effects model. For influencing factors, pooled odds ratios (ORs) with 95% confidence intervals (CIs) were estimated. Heterogeneity was assessed using Cochran’s Q test and the I^2^ statistic. Subgroup analyses, meta-regression, leave-one-out sensitivity analysis, and publication bias assessment were performed to evaluate the robustness of the findings.

**Results:**

A total of 48 studies involving 119,913,685 participants were included in the meta-analysis of screening uptake. The pooled uptake of upper gastrointestinal endoscopic screening was 38% (95% CI: 28–48%), with substantial heterogeneity. Subgroup analyses showed that screening uptake differed significantly by country (*p* = 0.047) and sample size (*p* = 0.001). Meta-regression indicated that publication year and sample size were not significant moderators of screening uptake (all *p* > 0.05). Sensitivity analysis demonstrated that the pooled estimate remained stable after sequential omission of each study, and Egger’s test did not indicate significant publication bias (*p* = 0.817). Among the 26 identified factors, 10 were included in the meta-analysis, and the remaining factors were summarized descriptively. The pooled results showed that female sex (OR = 1.16, 95% CI: 1.07–1.27), higher education (OR = 1.53, 95% CI: 1.35–1.74), occupational exposure to hazards (OR = 1.15, 95% CI: 1.08–1.23), drinking shallow water (OR = 1.32, 95% CI: 1.28–1.37), drinking deep well water (OR = 1.22, 95% CI: 1.05–1.40), a history of upper gastrointestinal diseases (OR = 1.94, 95% CI: 1.72–2.20), a family history of upper gastrointestinal cancer (OR = 1.50, 95% CI: 1.29–1.74), and a family history of cancer (OR = 1.57, 95% CI: 1.27–1.93) were associated with higher endoscopic screening uptake. In contrast, regular physical exercise (OR = 0.89, 95% CI: 0.81–0.97) and current smoking (OR = 0.86, 95% CI: 0.75–0.99) were associated with lower screening uptake.

**Conclusion:**

The uptake of upper gastrointestinal endoscopic screening remains suboptimal among screening-eligible populations. Screening participation appears to be influenced by socioeconomic factors, lifestyle factors, disease-related factors, cognitive factors, healthcare-related factors. These findings may inform targeted interventions to improve screening uptake and optimize the implementation of upper gastrointestinal cancer screening programs.

**Systematic review registration:**

https://www.crd.york.ac.uk/PROSPERO/view/CRD420261279918, identifier CRD420261279918.

## 1 Introduction

Upper gastrointestinal (GI) cancers, particularly gastric cancer (GC) and esophageal cancer (EC), contributed nearly 1.48 million incident cases and more than 1.10 million deaths globally (1). The global burden of upper GI cancers is geographically uneven, with East Asia consistently recognized as a high-incidence region for both esophageal cancer and gastric cancer (2). In 2022, there were an estimated 520,975 new gastric cancer cases and 248,384 new esophageal cancer cases, corresponding to age-standardized incidence rates of 15.1 and 6.0 per 100,000, respectively (3). Gastric cancer ranked third and esophageal cancer eighth among all newly diagnosed cancers in this region, highlighting the urgent need for earlier detection and timely clinical management.

Early detection is critical for improving the early prognosis of upper GI cancers (4). According to SEER data, the 5-year relative survival rate is 78.1% for localized gastric cancer but only 8.1% for distant-stage disease (5); similarly, it is 48.6% for localized esophageal cancer and 5.3% for distant-stage disease (6). Endoscopic screening can shift the diagnosis of upper GI cancers toward earlier stages by increasing the proportion of cancers detected at an early stage (7, 8). More importantly, accumulating evidence from East Asian populations suggests that endoscopic screening is associated with reduced mortality from upper GI cancers. A multicenter cohort study in China involving 637,500 residents aged 40–69 years from high-risk areas showed that participants who actually underwent one-time endoscopic screening had a 57% lower risk of upper GI cancer-related mortality than those in the control group (9) and it was further supported by a multicenter randomized controlled trial conducted in high-risk areas of China (10). In addition, evidence from the Korean National Cancer Screening Program (KNCSP) further showed that individuals who had ever undergone gastric cancer screening had lower odds of gastric cancer death than never-screened individuals, with the mortality reduction observed for upper endoscopy rather than upper GI series (11, 12). Besides, studies suggested that early detection of upper GI cancers could increase the overall survival and reduce the clinical and economic burden associated with advanced upper GI cancers (13, 14). These findings highlighted the importance of improving participation in endoscopic screening among individuals at eligible populations of upper GI cancers.

However, the effectiveness of screening programs depend not only on the efficacy of the screening test, but also on whether eligible individuals actually undergo the examination (15). Nevertheless, the pooled uptake of upper GI endoscopic screening and its associated factors across screening-eligible populations remained less synthesized. Against this background, this systematic review and meta-analysis aimed to synthesize the current uptake of upper GI endoscopic screening among populations eligible for upper GI endoscopic screening and to identify factors associated with upper GI cancer screening. By quantifying endoscopic uptake and summarizing its factors, this study seeks to provide an evidence base for future intervention development, improve the implementation efficiency of screening programs, and ultimately contribute to reducing the burden of upper GI cancer in East Asia.

## 2 Methods

The protocol for this systematic review and meta-analysis was registered in the PROSPERO with registration number CRD420261279918. This systematic review and meta-analysis followed the Preferred Reporting Items for Systematic Reviews and Meta-Analyses 2020 (PRISMA 2020) statement (16).

### 2.1 Search strategies

A comprehensive search was conducted across 8 databases: PubMed, Web of Science, Embase, CINAHL with full text, Scopus, CNKI, VIP and WanFang which covered literature from inception to May, 2026. Manual searches of the reference lists of relevant reviews were conducted to identify additional eligible studies. Combination of Medical Subject Headings and keywords were used to search. The full search strategy is showed in Supplementary Table S1.

### 2.2 Eligibility criteria

Studies were included if they met all of the following criteria: (1) the study population consisted of adults eligible for upper GI cancer screening, including either general target screening populations or high-risk populations. General target populations were defined as individuals within the age range recommended or covered by local screening programs, generally aged 40 years or older (17). High-risk populations were defined according to the original studies or screening programs, including residents of high-incidence areas, individuals identified as high risk through risk assessment or risk stratification, or those with relevant family history or other program-defined risk factors; (2) the study explicitly reported upper GI endoscopic screening uptake, defined as the proportion of the eligible, invited, or program-covered target population who completed upper GI endoscopy/gastroscopy for gastric cancer, esophageal cancer, or upper GI cancer screening; (3) sufficient numerator and denominator data were available for extraction or quantitative synthesis; and (4) the study was an original population-based observational screening study, including cross-sectional or cohort studies. Endoscopic screening uptake was calculated as follows: Endoscopic screening uptake (%) = Number of individuals who completed upper GI endoscopy / Eligible, invited, or program-covered target denominator population × 100%.

Studies were excluded if endoscopy was performed for diagnostic evaluation of symptoms, follow-up surveillance after previous upper GI disease or cancer, treatment monitoring, or other non-screening purposes. Studies were also excluded if they reported only the selection or distribution of screening modalities among individuals who had already participated in gastric cancer screening, or if they reported general gastric cancer screening participation without specifying endoscopy/gastroscopy-specific uptake. Studies in which the denominator included individuals outside the predefined screening age range were excluded unless age-restricted data for eligible participants could be extracted. Gray literature, theses, dissertations, randomized or cluster-randomized trials, reviews, conference abstracts, guidelines, letters, protocols, and commentaries were also excluded. Only articles published in Chinese or English were included.

### 2.3 Quality assessment

The methodological quality of the included studies was independently assessed by two reviewers (LYJ and HY). Any discrepancies were resolved through discussion or consultation with the third reviewer (ZY). For cross-sectional studies, the quality appraisal was conducted using the Agency for Healthcare Research and Quality (AHRQ) checklist (18). It contains 11 items, with each item scored as 1 for “Yes” and 0 for “No” or “Unclear.” Based on the total score, studies were classified as low quality (0–3 points), moderate quality (4–7 points), or high quality (8–11 points). We used the Newcastle-Ottawa Scale (NOS) (17) to assess cohort studies’ quality. The NOS included three domains: selection of the study population, comparability of study groups or participants, and assessment of outcomes, with a maximum score of 9 points. Studies scoring 0–3, 4–6, and 7–9 were considered low, moderate, and high quality, respectively.

### 2.4 Study selection and data extraction

Duplicate records were removed using EndNote X20. Two reviewers (TWH and PWY) independently screened the titles and abstracts and subsequently assessed the full texts of potentially eligible studies according to the predefined inclusion and exclusion criteria. Any discrepancies were resolved through discussion or consultation with a third reviewer (GYY). Data extraction was conducted independently by two reviewers (LYJ and XML) with unresolved disagreements adjudicated by a third reviewer (HY) using pre-designed extraction forms. The extracted information included the first author, year of publication, country, data collection period, study design, population, age eligibility, definition of endoscopic screening uptake, whether the screening program was government-led, sample size, cases, outcome reporting sources and associated factors. For the pooled estimation of upper GI endoscopic screening uptake, all eligible studies reporting both the number of participants who completed endoscopic screening and the corresponding eligible or invited denominator population were included, regardless of whether multivariable analyses were performed. In contrast, the meta-analysis of associated factors was restricted to studies that reported adjusted effect estimates, such as multivariable odds ratios, to reduce the influence of confounding.

### 2.5 Data synthesis and analysis

All statistical analyses were performed using Stata 18.0. A narrative synthesis was first conducted to summarize the characteristics of the included studies. When quantitative synthesis was feasible, meta-analysis was performed to estimate the pooled uptake of upper GI endoscopic screening and the pooled effects of factors associated with screening uptake.

The pooled uptake was synthesized as pooled proportions with corresponding 95% confidence intervals (CIs). The number of individuals who completed upper GI endoscopy was used as the numerator, and the eligible, invited, or program-covered target population was used as the denominator. The pooled uptake was calculated using the metaprop command in Stata 18.0 with a random-effects model, because substantial clinical and methodological heterogeneity was expected across studies. Exact binomial CIs were used for study-specific proportions using the cimethod (exact) option. The Stata command was: metaprop cases samplesize, random cimethod (exact) label (namevar = authoryear). Under the random-effects model, study weights were determined by both within-study variance and between-study variance; therefore, when between-study heterogeneity was high, the weights became more balanced across studies.

Factors associated with screening uptake were synthesized as odds ratios (ORs) with 95% CIs. Only factors reported in at least two studies with comparable classifications and consistent reference groups were considered eligible for meta-analysis. For these analyses, only adjusted effect estimates from multivariable models were extracted and pooled to reduce potential confounding bias. Adjusted ORs and their 95% CIs were log-transformed before pooling and then back-transformed for presentation.

Statistical heterogeneity across studies was assessed using Cochran’s *Q* test and the *I*^2^ statistic. Heterogeneity was considered non-significant when the *Q* test yielded a *p*-value greater than 0.05 and the *I*^2^ statistic was below 50%. To further examine potential sources of heterogeneity in the pooled estimate of screening uptake, subgroup analyses were performed according to country, outcome assessment source, study quality, study design, sample size, data collection duration, population type, government-led program status, and use of a two-step screening strategy. Meta-regression analyses were further performed using publication year and log-transformed sample size.

Sensitivity analysis was conducted using a leave-one-out approach to evaluate the robustness of the pooled estimate by sequentially omitting each study. A *post hoc* sensitivity analysis was also performed by excluding studies with fewer than 1,000 eligible, invited, or program-covered participants. Publication bias for the primary pooled estimate was assessed by visual inspection of the funnel plot and Egger’s test. A two-sided *p*-value < 0.05 was considered statistically significant.

## 3 Results

### 3.1 Search results

**Figure 1:**
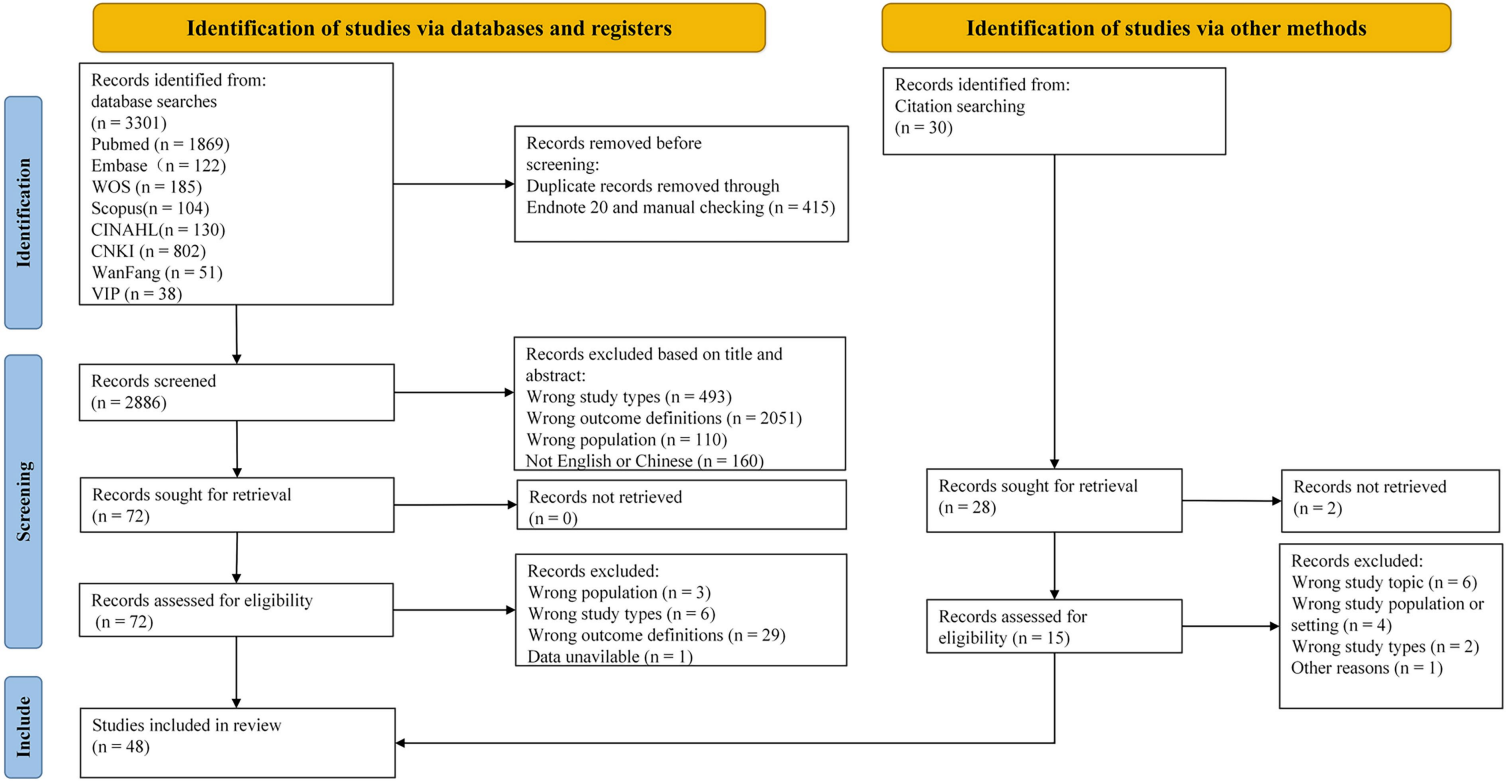
PRISMA flow diagram of the literature search and selection for studies.

The database search identified 3,301 records, and citation searching identified an additional 30 records. After duplicate removal, 2,886 records were screened by title and abstract. Subsequently, 72 records from databases and 28 records from citation searching were sought for full-text retrieval. After full-text assessment, 48 studies met the eligibility criteria and were included in the review. The PRISMA flowchart of the search and selection process was shown in Figure 1.

### 3.2 Characteristics of included studies

There are 36 cross-sectional studies and 12 cohort studies. According to the AHRQ assessment, 27 studies were rated as moderate quality and 9 as high quality (Supplementary Table S2). Based on the NOS, 2 cohort studies were rated as moderate quality and 10 as high quality (Supplementary Table S3). All studies were published within the period from 2008 to 2026. The majority of these studies were carried out in China (*n* = 38), followed by Korea (*n* = 8) and Japan (*n* = 2). The data collection periods ranged from 6 months to 17 years. Most studies had data collection periods longer than 6 years (*n* = 20), followed by >1–3 years and >3–6 years (*n* = 10 each), and ≤1 year (*n* = 7). Regarding program organization, the majority of studies were based on government-led screening programs (40/48, 80.0%), whereas only 8 studies were conducted outside government-led programs. Most studies obtained outcome data from standardized program records (*n* = 43), while only 5 studies relied on self-reported data.

### 3.3 Endoscopic uptake of upper GI cancer screening

**Figure 2:**
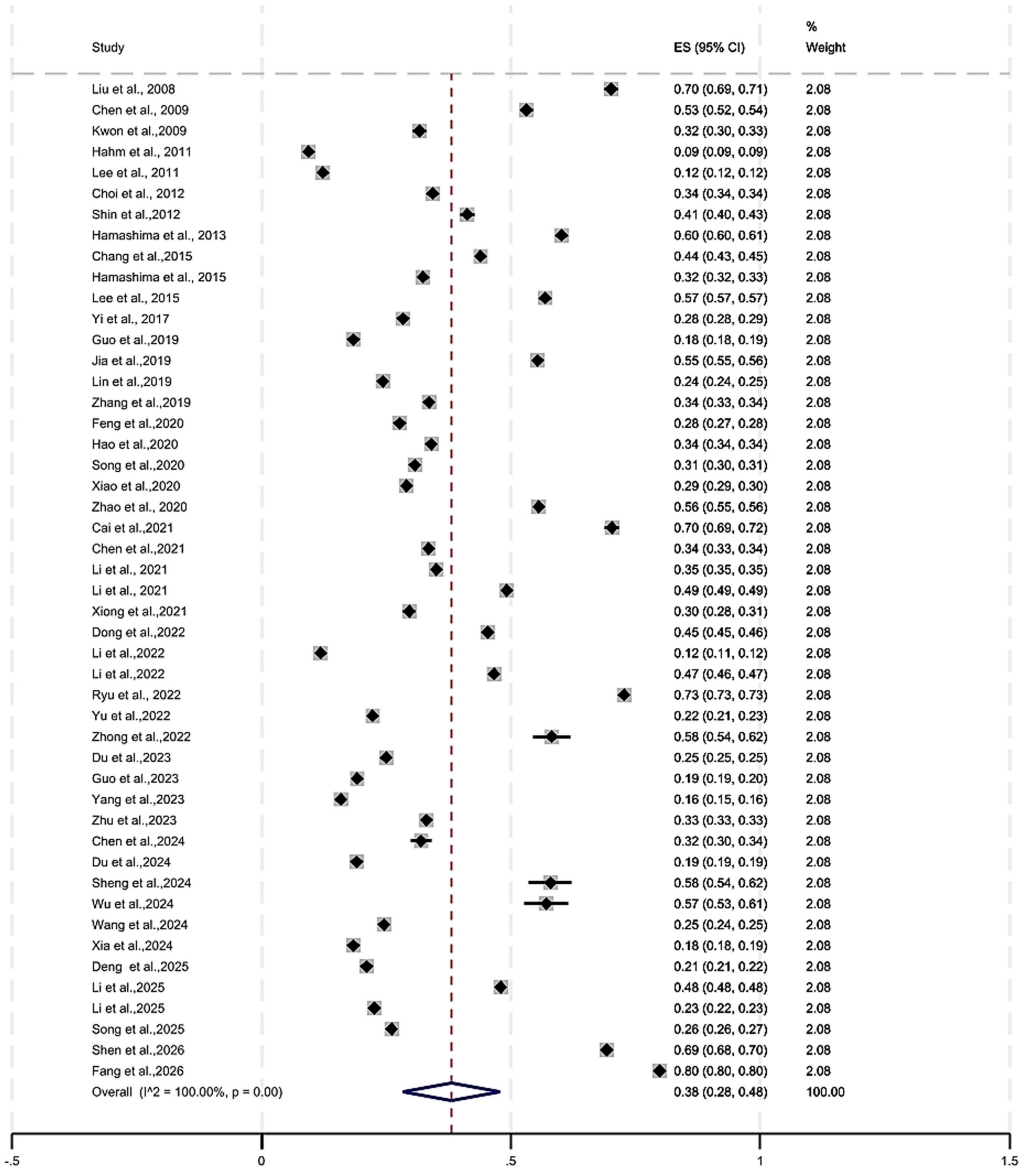
Forest plot of upper GI endoscopic screening uptake.

Across the included studies, the uptake rate of endoscopic screening for upper GI cancers ranged from 9.46 to 79.85% (Table 1). Given the heterogeneity among studies (*I*^2^ = 100%, *p* < 0.001), a random-effects model was used, which yielded a pooled uptake rate of 38.0% (95% CI: 28.0–48.0%, *p* < 0.001) (Figure 2).

**Table 1:** Basic characteristics of included studies.

Author, year	Country	Data collection duration	Study design	Population	Age eligible	Definition of endoscopic screening uptake	Sample size	Rate	Government-led program* (Yes/No)	Outcome sources	Influencing factors
Liu et al. (2008) (35)	China	2005–2006	Cross-Sectional Study	High-risk populations	40–69 years	Endoscopic screening uptake	5,010	70.10%	Yes	Standardized program records	/
Chen et al. (2009) (36)	China	2001.10–2002.10	Cohort study	High-risk populations	40–69 years	Endoscopic screening uptake	6,596	53.15%	No	Standardized program records	/
Kwon et al. (2009) (37)	Korea	2005	Cross-sectional study	General targeted population	≥40 years	Endoscopic screening uptake	4,593	31.71%	No	Self-reported	③④⑨㉑
Hahm et al. (2011) (38)	Korea	2005.01–2006.12	Cross-Sectional Study	General targeted population	≥40 years	Endoscopic screening uptake	22,913,618	9.46%	Yes	Standardized program records	/
Lee et al. (2011) (39)	Korea	2008.01–2008.12	Cross-Sectional Study	General targeted population	≥40 years	Endoscopic screening uptake	7,132,820	12.32%	Yes	Standardized program records	/
Choi et al. (2012) (40)	Korea	2002.01–2005.12	Cross-Sectional Study	General targeted population	≥40 years	Endoscopic screening uptake	2,690,731	34.37%	Yes	Standardized program records	/
Shin et al. (2012) (41)	Korea	2008	Cross-sectional study	General targeted population	≥40 years	Endoscopic screening uptake	4,464	41.30%	No	Self-reported	①③⑤⑧
Hamashima et al. (2013) (42)	Japan	2002.04–2007.03	Cohort study	General targeted population	40–79 years	Endoscopic screening uptake	42,236	60.16%	Yes	Standardized program records	/
Chang et al. (2015) (31)	Korea	2007–2009	Cross-sectional study	General targeted population	≥40 years	Endoscopic screening uptake	10,658	43.95%	No	Self-reported	①③④⑤⑥⑦⑧⑩⑮
Hamashima et al. (2015) (43)	Japan	2005–2010	Cohort study	General targeted population	40–79 years	Endoscopic screening uptake	50,521	32.41%	Yes	Standardized program records	/
Lee et al. (2015) (33)	Korea	2002–2011	Cross-Sectional Study	General targeted population	≥40 years	Endoscopic screening uptake	30,902,052	56.90%	Yes	Standardized program records	①②⑦
Yi et al. (2017) (44)	China	2000–2013	Cross-sectional study	General targeted population	40–69 years	Endoscopic screening uptake	206,105	28.42%	Yes	Standardized program records	/
Guo et al. (2019) (45)	China	2013.10–2017.10	Cross-Sectional Study	High-risk populations	40–74 years	Endoscopic screening uptake	43,423	18.40%	Yes	Standardized program records	①②③⑤⑨⑩⑪⑫⑬
Jia et al. (2019) (46)	China	2007–2016	Cross-Sectional Study	High-risk populations	40–69 years	Endoscopic screening uptake	28,543	55.39%	Yes	Standardized program records	⑭
Lin et al. (2019) (47)	China	2015–2018	Cross-sectional study	High-risk populations	40–74 years	Endoscopic screening uptake	25,300	24.34%	Yes	Standardized program records	/
Zhang et al. (2019) (48)	China	2009–2017	Cross-sectional study	High-risk populations	40–69 years	Endoscopic screening uptake	38,400	33.59%	Yes	Standardized program records	/
Feng et al. (2020) (49)	China	2006.10–2012.12	Cohort study	General targeted population	40–69 years	Endoscopic screening uptake	46,025	27.75%	Yes	Standardized program records	/
Hao et al. (2020) (50)	China	2005–2012	Cohort study	General targeted population	40–69 years	Endoscopic screening uptake	58,138	34.07%	Yes	Standardized program records	/
Song et al. (2020) (51)	China	2005–2013	Cohort study	General targeted population	40–69 years	Endoscopic screening uptake	71,923	30.78%	Yes	Standardized program records	/
Xiao et al. (2020) (52)	China	2012.10–2019.10	Cross-Sectional Study	High-risk populations	40–74 years	Endoscopic screening uptake	35,621	29.10%	Yes	Standardized program records	/
Zhao et al. (2020) (53)	China	2006–2015	Cohort study	General targeted population	40–69 years	Endoscopic screening uptake	42,188	55.63%	Yes	Standardized program records	/
Cai et al. (2021) (54)	China	2016.12–2019.12	Cross-Sectional Study	High-risk populations	40–69 years	Endoscopic screening uptake	3,400	70.30%	Yes	Standardized program records	/
Chen et al. (2021) (9)	China	2005–2012	Cohort study	High-risk populations	40–69 years	Endoscopic screening uptake	338,017	33.53%	Yes	Standardized program records	/
Li Jun et al. (2021) (55)	China	2006–2015	Cross-Sectional Study	High-risk populations	40–69 years	Endoscopic screening uptake	120,655	35.09%	Yes	Standardized program records	/
Li Jiang et al. (2021) (56)	China	2010–2016	Cross-Sectional Study	High-risk populations	40–69 years	Endoscopic screening uptake	276595.00	49.20%	Yes	Standardized program records	/
Xiong et al. (2021) (57)	China	2018–2019	Cross-Sectional Study	High-risk populations	40–74 years	Endoscopic screening uptake	5,365	29.60%	Yes	Standardized program records	/
Dong et al. (2022) (58)	China	2013–2018	Cross-Sectional Study	High-risk populations	40–69 years	Endoscopic screening uptake	70,927	45.36%	Yes	Standardized program records	①②③④⑤⑧⑨⑬⑯⑰
Li Q et al. (2022) (59)	China	2016.09–2020.03	Cross-sectional study	High-risk populations	40–69 years	Endoscopic screening uptake	16,862	11.89%	Yes	Standardized program records	①③⑧⑪⑫⑭⑯㉑㉔
Li H et al. (2022) (60)	China	2015–2017	Cross-Sectional Study	High-risk populations	40–69 years	Endoscopic screening uptake	23,532	46.62%	Yes	Standardized program records	Rural:①②③④⑧⑯⑰; Urban:①③④ ⑧⑯⑰
Ryu et al. (2022) (61)	Korea	2007–2016	Cross-Sectional Study	General targeted population	≥40 years	Endoscopic screening uptake	53,931,784	72.72%	Yes	Standardized program records	/
Yu et al. (2022) (62)	China	2016.10–2017.08	Cohort study	General targeted population	40–74 years	Endoscopic screening uptake	8,772	22.31%	Yes	Standardized program records	③⑧⑯⑰⑳㉔
Zhong et al. (2022) (26)	China	2020.10–2021.01	Cross-Sectional Study	High-risk populations	45–74 years	Endoscopic screening uptake	653	58.20%	No	Self-reported	③⑪㉒㉓
Du et al. (2023) (32)	China	2016–2022	Cross-Sectional Study	High-risk populations	40–74 years	Endoscopic screening uptake	47,748	25.02%	Yes	Standardized program records	①③⑥⑧⑫⑭⑯⑰⑳㉑
Guo et al. (2023) (63)	China	2013–2019	Cross-Sectional Study	High-risk populations	40–74 years	Endoscopic screening uptake	68,651	19.21%	Yes	Standardized program records	/
Yang et al. (2023) (64)	China	2014–2019	Cross-Sectional Study	High-risk populations	40–69 years	Endoscopic screening uptake	20,812	15.91%	Yes	Standardized program records	/
Zhu et al. (2023) (65)	China	2007–2021	Cross-Sectional Study	High-risk populations	40–69 years	Endoscopic screening uptake	78,191	33.12%	Yes	Standardized program records	/
Chen et al. (2024) (66)	China	2019–2020	Cross-Sectional Study	High-risk populations	45–74 years	Endoscopic screening uptake	1,792	32.00%	Yes	Standardized program records	/
Du et al. (2024) (67)	China	2012–2019	Cross-Sectional Study	High-risk populations	40–74 years	Endoscopic screening uptake	48,000	19.09%	Yes	Standardized program records	①③⑤⑧⑪⑫⑭⑮⑯ ⑰㉔
Sheng et al. (2024) (68)	China	2020.01–2022.12	Cross-Sectional Study	High-risk populations	≥40 years	Endoscopic screening uptake	500	58.00%	No	Self-reported	③④⑪⑯⑰㉒㉓
Wu et al. (2024) (69)	China	2023.01–2023.06	Cross-Sectional Study	High-risk populations	≥40 years	Endoscopic screening uptake	497	57.14%	No	Standardized program records	③⑨⑯⑰⑱⑲
Wang et al. (2024) (70)	China	2013–2019	Cohort study	High-risk populations	40–74 years	Endoscopic screening uptake	39,163	24.60%	Yes	Standardized program records	/
Xia et al. (2024) (71)	China	2017.10–2022.10	Cross-Sectional Study	High-risk populations	40–75 years	Endoscopic screening uptake	18,500	18.42%	No	Standardized program records	①②③⑤⑧⑨⑩⑯⑰
Deng et al. (2025) (72)	China	2023	Cross-sectional study	High-risk populations	40–74 years	Endoscopic screening uptake	18,494	21.10%	Yes	Standardized program records	/
Li et al. (2025) (73)	China	2007–2023	Cross-sectional study	High-risk populations	40–69 years (before 2022); 45–74 years (after 2022)	Endoscopic screening uptake	168,964	47.96%	Yes	Standardized program records	①②④⑤⑧⑩⑬ ㉑
Li et al. (2025) (34)	China	2013–2022	Cohort study	High-risk populations	45–74 years	Endoscopic screening uptake	51,888	22.64%	Yes	Standardized program records	⑫⑮⑯⑰⑳㉕㉖
Song et al. (2025) (74)	China	2014–2015	Cohort study	High-risk populations	40–69 years	Endoscopic screening uptake	39,991	26.10%	Yes	Standardized program records	③⑯⑰
Fang et al. (2026) (75)	China	2007–2023	Cross-sectional study	High-risk populations	40–74 years	Endoscopic screening uptake	140,338	79.85%	Yes	Standardized program records	/
Shen et al. (2026) (76)	China	2022–2024	Cross-sectional study	High-risk populations	40–75 years	Endoscopic screening uptake	14,629	69.23%	Yes	Standardized program records	/

*Studies conducted within national or local early detection and treatment demonstration programs, organized cancer screening programs, or government-supported public health screening projects were coded as government-led programs. Influencing factors: ① Age; ② Gender; ③ Education; ④ Income level; ⑤ Marital status; ⑥ Employment status; ⑦ Health insurance; ⑧ Smoking exposure; ⑨ Drinking; ⑩ BMI/Obesity; ⑪ Physical activity; ⑫ Dietary habits; ⑬ Untreated/non-tap water exposure; ⑭ Household cooking fumes; ⑮ Occupational exposure; ⑯ History of upper GI/digestive system disease; ⑰ History of upper GI cancer; ⑱ *Helicobacter pylori* infection; ⑲ History of gastroscopy; ⑳ Comorbidities/general health status; ㉑ Family history of cancer; ㉒ Alarm symptoms; ㉓ Cognitive factors; ㉔ Psychological trauma or depression; ㉕ Screening publicity or mobilization; ㉖ Screening support resources.

### 3.4 Subgroup analysis and meta-regression analysis

Subgroup analyses showed significant differences by country and sample size (Table 2), and the corresponding forest plots are presented in Supplementary Figure S2. By country, the pooled uptake was 0.38 in China (95% CI: 0.32–0.43), 0.38 in Korea (95% CI: 0.14–0.62), and 0.44 in Japan (95% CI: 0.44–0.45), with a significant subgroup difference (*p* = 0.047). A significant difference was also observed across sample-size categories (*p* = 0.001), with pooled uptake ranging from 0.16 to 0.58.

**Table 2:** Subgroup analyses of pooled uptake of upper GI endoscopic screening.

Subgroup variable	Category	No. of studies	Pooled uptake (95% CI)	*I*^2^ (%)	*P* for within-subgroup heterogeneity	*P* for subgroup difference
Overall	Total	48	0.38 (0.28–0.48)	100.00%	<0.001	–
Country	China	38	0.38 (0.32, 0.43)	100.00	<0.001	0.047
	Korea	8	0.38 (0.14–0.62)	99.99	<0.001	
	Japan	2	0.44 (0.44–0.45)	–	–	
Outcome assessment	Self-reported	5	0.46 (0.39–0.53)	98.88	<0.001	0.140
	Standardized program records	43	0.37 (0.27–0.47)	100.00	<0.001	
Study design	Cross-sectional study	36	0.39 (0.28–0.50)	99.96	<0.001	0.560
	Cohort study	12	0.35 (0.30–0.41)	100.00	<0.001	
Study quality	High	19	0.34 (0.20–0.48)	100.00	<0.001	0.516
	Moderate	29	0.41 (0.26–0.56)	100.00	<0.001	
Sample size	10^2^	3	0.58 (0.55–0.60)	98.80	<0.001	0.001
	10^3^	8	0.44 (0.30–0.57)	99.50	<0.001	
	10^4^	26	0.32 (0.28–0.37)	100.00	<0.001	
	10^5^	6	0.46 (0.31–0.61)	100.00	<0.001	
	10^6^	2	0.16 (0.16–0.16)	–	<0.001	
	10^7^	3	0.46 (0.05–0.88)	–	<0.001	
Data collection duration	≤1 year	7	0.35 (0.27–0.43)	99.97	<0.001	0.484
	>1–3 years	10	0.44 (0.28–0.60)	99.99	<0.001	
	>3–6 years	10	0.33 (0.26–0.41)	99.98	<0.001	
	>6 years	21	0.39 (0.33–0.44)	100.00	<0.001	
Population type	General target population	16	0.37 (0.20–0.54)	98.86	<0.001	0.880
	High-risk population	32	0.39 (0.32–0.45)	99.95	<0.001	
Government-led program	Yes	40	0.45 (0.33–0.57)	100.00	<0.001	0.280
	No	8	0.36 (0.26–0.47)	100.00	<0.001	
Two-step screening strategies	Yes	28	0.35 (0.28–0.43)	99.00	<0.001	0.410
	No	20	0.42 (0.27–0.52)	100.00	<0.001	

All pooled estimates were calculated using a random-effects model. *P* for within-subgroup heterogeneity refers to the heterogeneity test conducted within each subgroup. *P* for subgroup difference refers to the test for differences between subgroups. Abbreviations: CI, confidence interval.

No significant subgroup differences were observed by outcome assessment source, study design, study quality, data collection duration, population type, or government-led program status. The pooled uptake was 0.46 for self-reported outcomes and 0.37 for standardized program records (*p* = 0.140). Cross-sectional studies had a pooled uptake of 0.39, whereas cohort studies had a pooled uptake of 0.35 (*p* = 0.560). The pooled uptake was 0.34 in high-quality studies and 0.41 in moderate-quality studies (*p* = 0.516). By data collection duration, uptake ranged from 0.33 to 0.44 across subgroups, with no significant difference (*p* = 0.484). Uptake was also comparable between general target populations and high-risk populations (0.37 vs. 0.39, *p* = 0.880). For government-led program status, the pooled uptake was 0.45 among government-led studies and 0.36 among non-government-led studies, but the subgroup difference was not statistically significant (*p* = 0.280). Substantial heterogeneity remained within most subgroups (Table 3). Meta-regression analyses indicated that publication year (*β* = −0.0008, 95% CI: −0.0117–0.0101, *p* = 0.880) and log-transformed sample size (*β* = −0.0086, 95% CI: −0.0299 – 0.0126, *p* = 0.417) were not significantly associated with screening uptake, suggesting that these covariates did not explain the observed heterogeneity.

**Table 3:** Results of the meta regression analyses.

Covariate	*β*	SE	95%CI	*P*
Publication year	−0.0008	0.0054	−0.0117-0.0101	0.880
Sample size (ln)	−0.0086	0.0105	−0.0299-0.0126	0.417

β, regression coefficient; SE, standard error; CI, confidence interval.

### 3.5 Associated factors of upper GI cancer screening

A total of 26 factors associated with upper GI cancer screening uptake were descriptively summarized in Table 1. These factors were categorized into four dimensions: socioeconomic factors (*n* = 7), lifestyle-related factors (*n* = 8), disease-related factors (*n* = 8), and cognitive and healthcare-related factors (*n* = 3). Socioeconomic factors included age, sex, education, income level, marital status, employment status, and health insurance. Lifestyle-related factors included smoking, alcohol drinking, BMI/obesity, physical activity, dietary factors, untreated or non-tap water exposure, household cooking fumes, and occupational exposure. Disease-related factors included a history of upper GI or digestive system disease, a history of upper GI cancer, *Helicobacter pylori* infection, a history of gastroscopy, comorbidities or general health status, a family history of cancer, alarm symptoms, and psychological trauma or depression. Cognitive and healthcare-related factors included cognitive factors, screening publicity or mobilization, and screening support resources.

Of the 26 factors, only 10 were eligible for meta-analysis, yielding 12 pooled comparisons. The pooled numerical results are summarized in Table 4, the corresponding forest plots are presented in Supplementary Figure S3, and the study-level estimates underlying these analyses are provided in Supplementary Table S4. The remaining factors were not quantitatively synthesized because they were not comparable across studies due to substantial classification heterogeneity, inconsistent reference groups, or insufficient data. As shown in Table 4, women were more likely to undergo upper GI endoscopic screening than men (OR = 1.16, 95% CI: 1.07–1.27, *p* < 0.001). Individuals with higher education also had a higher likelihood of screening uptake than those with lower education (OR = 1.53, 95% CI: 1.35–1.74, *p* < 0.001). Compared with individuals without regular physical exercise, those who reported regular physical exercise were less likely to undergo screening (OR = 0.89, 95% CI: 0.81–0.97, *p* = 0.008). For smoking status, ex-smokers showed no significant difference in screening uptake compared with never smokers (OR = 1.04, 95% CI: 0.89–1.22, *p* = 0.615), whereas current smokers were less likely to participate in screening (OR = 0.86, 95% CI: 0.75–0.99, *p* = 0.032). Participants with occupational exposure to hazards were more likely to undergo screening than those without such exposure (OR = 1.15, 95% CI: 1.08–1.23, *p* < 0.001). Compared with treated tap water, drinking shallow water (OR = 1.32, 95% CI: 1.28–1.37, *p* < 0.001) and drinking deep well water (OR = 1.22, 95% CI: 1.05–1.40, *p* = 0.009) were both associated with higher screening uptake. Marital status was not significantly associated with screening uptake (married/with spouse vs. unmarried/divorced/widowed: OR = 1.04, 95% CI: 0.88–1.23, *p* = 0.675). In addition, participants with a history of upper GI diseases (OR = 1.94, 95% CI: 1.72–2.20, *p* < 0.001), a family history of upper GI cancer (OR = 1.50, 95% CI: 1.29–1.74, *p* < 0.001), or a family history of cancer (OR = 1.57, 95% CI: 1.27–1.93, *p* < 0.001) were significantly more likely to undergo screening. Substantial heterogeneity was observed for most pooled comparisons.

**Table 4:** Meta-analysis results of factors associated with upper GI endoscopic screening uptake.

Influencing factors	Comparison	Control	Number of studies	Heterogeneity		Model	OR (95%CI)	*p*-value
				*I*^2^	*P*-value			
Gender	Female	Men	10 (25, 29, 31, 44, 46, 48, 55, 59, 61, 63)	95.2%	*P* < 0.001	Random	1.16 (1.07–1.27)	*P* < 0.001
Education	Higher education level	Lower Education level	15 (21, 25, 27, 31, 44–46, 48–50, 55–57, 59, 63)	92.3%	*p* < 0.001	Random	1.53 (1.35–1.74)	*p* < 0.001
Regular physical exercise	Yes	No	7 (25, 31, 45, 49, 55, 56, 62)	83.5%	*P* < 0.001	Random	0.89 (0.81–0.97)	*P* = 0.001
Smoking	Ex-smoker	Never	8 (21, 25, 31, 44, 46, 50, 55, 59)	94.3%	*P* < 0.001	Random	1.04 (0.89–1.22)	*p* = 0.615
	Current smoker		9 (21, 25, 27, 31, 45, 50, 55, 59, 61)	97.2%	*P* < 0.001	Random	0.86 (0.75–0.99)	*p* = 0.032
Occupational exposure to hazards	Yes	No	2 (55, 62)	69.5%	*P* < 0.001	Random	1.15 (1.08–1.23)	*P* < 0.001
Drinking untreated water sources	Shallow water	Tap water (treated)	2 (44, 61)	–	*P* < 0.001	Fixed	1.32 (1.28–1.37)	*P* < 0.001
	Deep well water		2 (44, 61)	96.5%	*P* < 0.001	Random	1.22(1.05–1.40)	*p* = 0.009
Marriage status	Married/with spouse	Unmarried/divorced/widowed	8 (21, 25, 27, 31, 55, 59, 61, 63)	92.20%	*P* < 0.001	Random	1.04(0.88–1.23)	*p* = 0.675
History of upper GI diseases	Yes	No	8 (27, 31, 48, 50, 55–57, 63)	92.10%	*P* < 0.001	Random	1.94 (1.72–2.20)	*P* < 0.001
Family history of UGC	Yes	No	9 (31, 44, 46, 48, 50, 55, 57, 59, 62)	97.2%	*P* < 0.001	Random	1.50(1.29–1.74)	*P* < 0.001
Family history of cancer	Yes	No	6 (45, 56, 57, 61–63)	99.0%	*P* < 0.001	Random	1.57 (1.27–1.93)	*P* < 0.001

### 3.6 Sensitivity analysis and publication bias

A leave-one-out sensitivity analysis showed that the pooled estimates remained stable, ranging from 0.37 to 0.39 after sequential omission of each study, suggesting that no single study had a disproportionate influence on the overall pooled estimate (Supplementary Figure S1). Funnel plot inspection (Supplementary Figure S4) and Egger’s test did not detect statistically significant asymmetry (*p* = 0.817).

## 4 Discussion

This systematic review and meta-analysis provides a comprehensive synthesis of the uptake of upper GI endoscopic screening and its associated factors in East Asian populations. Despite the disproportionate burden of gastric cancer and esophageal cancers in East Asia (1, 19), the pooled uptake of upper GI endoscopic screening was only 38.0%, indicating that approximately two-thirds of eligible individuals had not undergone endoscopic examination. The low uptake observed in this study highlights a persistent implementation gap between screening efficacy and real-world screening strategies.

The pooled endoscopic screening uptake was 38% in China (*n* = 38), 38% in Korea (*n* = 8), and 44% in Japan (*n* = 2), suggesting possible cross-country variation in endoscopic screening participation. This finding is broadly consistent with a recent systematic review of gastric cancer screening programs (20), which also reported country-level differences in endoscopic uptake and found higher uptake in Japan and Korea than in China (21). A significant subgroup difference was also observed by sample size. Studies with smaller sample sizes tended to report higher uptake estimates, whereas larger studies, particularly those based on large-scale program records, generally yielded more conservative estimates. This pattern may reflect differences in study setting, denominator definition, implementation intensity, and data source. Smaller studies may be conducted in selected communities or pilot programs with stronger mobilization, while large program-based studies may better capture real-world participation across broader populations. However, meta-regression using log-transformed sample size did not show a significant linear association with uptake, suggesting that the subgroup finding by sample size should be interpreted as exploratory rather than definitive.

No significant subgroup differences were observed by outcome assessment source, study design, study quality, data collection duration, population type, or government-led program status. Although self-reported studies showed a higher pooled uptake than studies using standardized program records, the difference was not statistically significant. Nevertheless, self-reported outcomes may still be affected by recall bias and broader interpretation of previous endoscopy history, whereas standardized program records are more likely to capture actual completion within a defined screening pathway. Similarly, cross-sectional studies showed a slightly higher pooled uptake than cohort studies, but the subgroup difference was not significant. This suggests that study design alone did not fully explain the observed heterogeneity in the updated analysis. Government-led studies also showed a higher pooled uptake than non-government-led studies, but the subgroup difference was not statistically significant. This finding may indicate that government-led programs alone are insufficient to guarantee high participation unless accompanied by effective invitation, risk communication, appointment coordination, financial support, and follow-up mechanisms. In addition, meta-regression analyses showed that neither publication year nor log-transformed sample size was significantly associated with screening uptake, suggesting that these covariates did not explain the substantial between-study heterogeneity.

Among the 26 identified influencing factors, only 10 were eligible for meta-analysis, yielding 12 pooled comparisons. The remaining factors were not quantitatively synthesized because of substantial heterogeneity in classification, inconsistent reference groups, or insufficient data. The pooled results showed that women were more likely to undergo upper GI endoscopic screening than men. This finding is consistent with results from other analyses of factors influencing screening participation, which may be attributed to women having more opportunities for medical contact throughout their life cycle and stronger health prevention awareness (22, 23). Higher education was also associated with greater screening uptake, consistent with previous studies (22, 24). Individuals (15, 25) with higher educational levels tend to have higher health literacy, leading to more frequent engagement in cancer prevention behaviors. These mechanisms may be particularly important for endoscopic screening, as endoscopy is invasive, requires preparation, and is often associated with concerns about discomfort, cost, and potential complications. Therefore, individuals with lower educational levels should be provided with more cancer prevention knowledge, accessible health education, clearer risk communication, and stronger navigation support.

Several disease-related factors were also associated with higher screening uptake. Participants with a history of upper GI (GI) diseases, a family history of upper GI cancer, or a family history of other cancers were more likely to undergo screening. These factors may increase individuals’ perceived susceptibility to and severity of the disease, thereby motivating them to seek endoscopic examinations. This aligns with the Health Belief Model, and the findings of Zhong et al. (26) have also validated this view. Additionally, a history of upper GI disease, family history of upper GI cancer, or other cancers are established risk factors for upper GI cancers (27, 28). Individuals with these backgrounds face a higher risk of developing symptoms related to upper GI cancer, making them more willing to undergo screening (29). Occupational exposure to hazards (*n* = 2) was also positively associated with screening uptake. This may partially reflect heightened risk awareness among exposed individuals or the inclusion of occupational and environmental exposures as criteria in some high-risk screening programs. Similarly, drinking untreated water sources including shallow water (*n* = 2) and deep well water (*n* = 2) was associated with higher screening uptake compared to consuming treated tap water. These findings suggested that future gastric cancer prevention and control strategies should continue to strengthen risk-stratified screening models, using occupational and environmental exposures as effective tools to identify high-risk groups. Moreover, precise health education and accessible screening services should be implemented to translate such risk awareness into actual preventive behaviors. However, due to the limited number of studies available, these results require cautious interpretation.

In contrast, regular physical exercise and current smoking were both associated with lower screening uptake. While numerous studies (30) have shown that regular physical exercise can reduce cancer risk, and the necessity of early diagnosis for upper GI cancers has been fully established due to the insidious onset. Future research should further explore why individuals who engage in regular physical activity have low screening participation rates. This will enable the development of targeted attention and intervention strategies. The negative association between current smoking and screening uptake is noteworthy because smoking is a recognized risk factor for upper GI cancers. This finding suggests that current smokers may be less engaged in preventive healthcare and should be considered a priority group for targeted screening promotion. Former smoking was not significantly associated with screening uptake in the updated pooled analysis. It suggests that individuals with healthier lifestyles may not necessarily perceive themselves as needing endoscopic screening, especially when they do not have symptoms or known risk factors. Marital status was not significantly associated with screening uptake in the updated meta-analysis, indicating that the role of family or spousal support may vary across populations and screening contexts.

For the remaining factors, descriptive summary was made (Table 1). Age and income level were frequently identified as factors associated with endoscopic screening uptake. Specifically, 10 studies reported age as a significant determinant, and six studies reported a significant association with income level. However, because the categorization of age and income varied substantially across the included studies, these variables could not be quantitatively synthesized in the meta-analysis. Nevertheless, the direction of association was generally consistent across studies, suggesting that older individuals and those with higher income levels were more likely to participate in endoscopic screening. Employment status was less frequently examined as a determinant of endoscopic screening uptake, with only two studies reporting relevant findings (31, 32). One study reported that, compared with office employees, farmers had a higher likelihood of participating in endoscopic screening, whereas workers showed a lower screening uptake. In contrast, another study found that employed individuals were less likely to undergo endoscopic screening than those without employment. These inconsistent findings may be partly attributable to differences in occupational classification, study populations, screening settings, and local program implementation. Given the limited number of studies and the heterogeneity in exposure definitions, the association between employment status and endoscopic screening uptake should be interpreted cautiously and warrants further investigation. Health insurance coverage was identified as a potential facilitator of endoscopic screening uptake. Studies by Lee et al. (33) and Chang et al. (31) reported that individuals covered by health insurance were more likely to participate in endoscopic screening, suggesting that financial protection and reimbursement mechanisms may reduce barriers to screening participation. In addition, alcohol consumption and obesity were reported as significant factors associated with screening uptake in four and two studies, respectively. However, the direction and magnitude of these associations were inconsistent across studies, and the limited number of available studies precluded a quantitative synthesis. Further research is therefore needed to clarify the roles of alcohol consumption and obesity in endoscopic screening participation. Several studies reported associations between endoscopic screening uptake and dietary habits or exposure to cooking fumes. Individuals with unhealthy dietary habits and frequent exposure to cooking fumes tended to show higher screening participation, possibly reflecting greater perceived risk among those exposed to recognized risk-related factors. However, further studies are needed to confirm these associations. The provision of additional support within screening programs was also identified as a potential facilitator of endoscopic screening uptake. However, only one study (34) reported this factor, and the evidence remains limited. Future studies should give greater consideration to external and program-level determinants of screening participation.

This study has several strengths. First, it focused on East Asia, a region where upper GI cancers remain a major public health concern and where endoscopic screening is highly relevant for early detection. Second, this review synthesized not only pooled screening uptake but also a broad range of associated factors, providing a more comprehensive understanding of endoscopic screening participation. Third, subgroup analyses and meta-regression were conducted to explore potential sources of heterogeneity, including study design, outcome assessment source, study quality, population type, and government-led program status. These analyses helped clarify which study-level characteristics were associated with variation in pooled uptake estimates.

Several limitations should also be acknowledged. First, substantial heterogeneity persisted across most analyses, reflecting differences in screening definitions, target populations, denominator selection, screening pathways, healthcare systems, and data sources. Therefore, the pooled uptake estimate should be interpreted as a broad summary of available evidence rather than a single uniform estimate applicable to all settings. Second, most included studies were observational, and many were cross-sectional, limiting causal inference regarding factors associated with screening uptake. Third, the evidence base was geographically imbalanced, with most studies conducted in China and fewer studies from Korea and Japan. The findings should therefore not be interpreted as fully representative of all East Asian populations. Fourth, some included studies did not clearly distinguish preventive screening endoscopy among asymptomatic individuals from diagnostic endoscopy prompted by symptoms. This may have led to overestimation of true asymptomatic screening uptake, although it may also reflect real-world early detection examination behavior. Fifth, many influencing factors could not be pooled because of inconsistent definitions, heterogeneous classifications, and different reference groups. Future studies should adopt standardized definitions of endoscopic screening uptake, clearly distinguish screening from diagnostic endoscopy, and use harmonized measures for behavioral and healthcare-related determinants.

In conclusion, this study showed that upper GI endoscopic screening uptake in East Asia remains suboptimal. Screening participation was associated with multiple socioeconomic, lifestyle-related, disease-related, cognitive, and healthcare-related factors. These findings may help identify populations with lower screening participation and inform future intervention strategies, including risk-stratified invitation, tailored health education, physician recommendation, barrier reduction, reminder systems, and system-level support to improve completion of upper GI endoscopic screening.

## Data Availability

The original contributions presented in the study are included in the article/Supplementary material, further inquiries can be directed to the corresponding authors.
