# Supplementary material for: Upper gastrointestinal endoscopic screening uptake and associated factors in East Asia: a systematic review and meta-analysis
Source: Front Public Health. 2026 Jul 29;14:1869526. doi: 10.3389/fpubh.2026.1869526 (PMC13461455; doi:10.3389/fpubh.2026.1869526)
Supplement: Supplementary file 1 [file Data_Sheet_1.pdf]

Supplementary Materials Contents

Supplementary Table S1. Search strategies of the databases
Supplementary Table S2. Quality assessment of cross-sectional studies using the Agency for Healthcare Research and Quality (AHRQ) checklist
Supplementary Table S3. Quality assessment of cohort studies using the Newcastle–Ottawa scale (NOS)
Supplementary Table S4. Study-level estimates included in the meta-analysis of associated factors
Supplementary Figure S1. Sensitivity analysis for meta-analyses of pooled participation rates
Supplementary Figure S2. Forest plots of subgroup analyses for upper gastrointestinal endoscopic screening uptake
Supplementary Figure S3. Forest plots of factors associated with upper gastrointestinal endoscopic screening uptake
Supplementary Figure S4. Funnel plot and egger's test of effect sizes against standard errors to assess publication bias

Table S1. Search strategies of the databases

Database	PubMed (1,869)
URL	https://www.ncbi.nlm.nih.gov/pubmed/
Time	Inception to 2026.5
Search strategy	#1 "Gastrointestinal Neoplasms"[MeSH Terms] OR "Stomach Neoplasms"[MeSH Terms]<br>#2 "upper gastrointestinal cancer*"[Title/Abstract] OR "upper gastrointestinal neoplasm*"[Title/Abstract] OR "upper gastrointestinal tract cancer*"[Title/Abstract] OR "upper digestive tract cancer*"[Title/Abstract] OR "upper digestive tract neoplasm*"[Title/Abstract] OR "esophageal cancer"[Title/Abstract] OR "oesophageal cancer"[Title/Abstract] OR "esophageal neoplasm*"[Title/Abstract] OR "oesophageal neoplasm*"[Title/Abstract] OR "gastric cancer"[Title/Abstract] OR "stomach cancer"[Title/Abstract] OR "gastric neoplasm*"[Title/Abstract] OR "stomach neoplasm*"[Title/Abstract]<br>#3 ("endoscopic screening"[Title/Abstract] OR "endoscopy screening"[Title/Abstract] OR "screening endoscopy"[Title/Abstract] OR "upper gastrointestinal endoscopy"[Title/Abstract] OR "upper GI endoscopy"[Title/Abstract] OR "gastroscopy"[Title/Abstract] OR "screening gastroscopy"[Title/Abstract] OR "esophagogastroduodenoscopy"[Title/Abstract] OR "EGD"[Title/Abstract])<br>#4 ("participation"[Title/Abstract] OR "uptake"[Title/Abstract] OR "attendance"[Title/Abstract] OR "adherence"[Title/Abstract] OR "compliance"[Title/Abstract] OR "intention"[Title/Abstract] OR "willingness"[Title/Abstract] OR "acceptability"[Title/Abstract])<br>#5 "barrier*"[Title/Abstract] OR "facilitator*"[Title/Abstract] OR "obstacle*"[Title/Abstract] OR "challenge*"[Title/Abstract] OR "enabler*"[Title/Abstract] OR "determinant*"[Title/Abstract] OR "predictor*"[Title/Abstract] OR "factor*"[Title/Abstract] OR "influence*"[Title/Abstract] OR "views*"[Title/Abstract] OR "Awareness"[Title/Abstract] OR "perspective*"[Title/Abstract] OR "perception"[Title/Abstract] OR "opinion*"[Title/Abstract] OR "experience*"[Title/Abstract] OR "challenge*"[Title/Abstract]<br>#6 (#1 OR #2) AND #3 AND (#4 OR #5)

Database	Embase (122)
URL	https://www.embase.com/
Time	Inception to 2026.5
Search strategy	#1 'stomach tumor'/exp OR 'esophagus tumor'/exp<br>#2 'upper gastrointestinal cancer*':ti,ab,kw OR 'upper gastrointestinal neoplasm*':ti,ab,kw OR 'upper gastrointestinal tract cancer*':ti,ab,kw OR 'upper digestive tract cancer*':ti,ab,kw OR 'upper digestive tract neoplasm*':ti,ab,kw OR 'esophageal cancer':ti,ab,kw OR 'oesophageal cancer':ti,ab,kw OR 'esophageal neoplasm*':ti,ab,kw OR 'oesophageal neoplasm*':ti,ab,kw OR 'gastric cancer':ti,ab,kw OR 'stomach cancer':ti,ab,kw OR 'gastric neoplasm*':ti,ab,kw OR 'stomach neoplasm*':ti,ab,kw<br>#3 'endoscopic screening':ti,ab,kw OR 'endoscopy screening':ti,ab,kw OR 'screening endoscopy':ti,ab,kw OR 'upper gastrointestinal endoscopy':ti,ab,kw OR 'upper gi endoscopy':ti,ab,kw OR 'screening gastroscopy':ti,ab,kw OR ((screen* NEAR/5 endoscop*) OR (screen* NEAR/5 gastroscop*) OR (screen* NEAR/5 esophagogastroduodenoscop*) OR (screen* NEAR/5 egd)):ti,ab,kw<br>#4 'screening uptake':ti,ab,kw OR 'screening participation':ti,ab,kw OR 'screening attendance':ti,ab,kw OR 'screening adherence':ti,ab,kw OR 'screening compliance':ti,ab,kw OR 'participation rate':ti,ab,kw OR 'uptake rate':ti,ab,kw OR 'attendance rate':ti,ab,kw OR 'screening behavior':ti,ab,kw OR 'screening behaviour':ti,ab,kw OR 'screening utilization':ti,ab,kw OR 'screening utilisation':ti,ab,kw OR ((participation OR uptake OR attendance OR adherence OR compliance OR intention OR willingness OR acceptability) NEAR/5 (screen* OR endoscop* OR gastroscop* OR esophagogastroduodenoscop* OR egd)):ti,ab,kw<br>#5 ((barrier* OR facilitator* OR obstacle* OR enabler* OR determinant* OR predictor* OR factor* OR influence*) NEAR/5 (uptake OR participation OR attendance OR adherence OR compliance OR intention OR willingness OR acceptability)):ti,ab,kw OR ((view* OR awareness OR perspective* OR perception* OR opinion* OR experience* OR challenge*) NEAR/5 (screen* OR endoscop* OR gastroscop* OR esophagogastroduodenoscop* OR egd)):ti,ab,kw<br>#6 #1 OR #2<br>#7 #4 OR #5<br>#8 #3 AND #6 AND #7

Database	Scopus (104)
URL	https://www.scopus.com/pages/home#basic
Time	Inception to 2026.5
Search strategy	TITLE-ABS-KEY(("upper gastrointestinal cancer*" OR "upper gastrointestinal neoplasm*" OR "upper gastrointestinal tract cancer*" OR "upper digestive tract cancer*" OR "upper digestive tract neoplasm*" OR "esophageal cancer" OR "oesophageal cancer" OR "esophageal neoplasm*" OR "oesophageal neoplasm*" OR "gastric cancer" OR "stomach cancer" OR "gastric neoplasm*" OR "stomach neoplasm*"))<br>AND TITLE-ABS-KEY(("endoscopic screening" OR "endoscopy screening" OR "screening endoscopy" OR "upper gastrointestinal endoscopy" OR "upper GI endoscopy" OR "screening gastroscopy" OR ((screen* W/5 endoscop*) OR (screen* W/5 gastroscop*) OR (screen* W/5 esophagogastroduodenoscop*) OR (screen* W/5 EGD))))<br>AND TITLE-ABS-KEY(("screening uptake" OR "screening participation" OR "participation rate" OR "uptake rate" OR "screening attendance" OR "screening adherence" OR "screening compliance" OR ((uptake OR participation OR attendance OR adherence OR compliance OR intention OR willingness OR acceptability) W/5 (screen* OR endoscop* OR gastroscop* OR esophagogastroduodenoscop* OR EGD)) OR ((barrier* OR facilitator* OR obstacle* OR determinant* OR predictor* OR "associated factor*" OR "influencing factor*") W/5 (uptake OR participation OR attendance OR adherence OR compliance OR intention OR willingness OR acceptability OR screen*))))

Database	Web of Science Core Collection (185)
URL	https://webofscience.clarivate.cn/wos/woscc/smart-search
Time	Inception to 2026.5
Search strategy	#1 TS=("upper gastrointestinal cancer*" OR "upper gastrointestinal neoplasm*" OR "upper gastrointestinal tract cancer*" OR "upper digestive tract cancer*" OR "upper digestive tract neoplasm*" OR "esophageal cancer" OR "oesophageal cancer" OR "esophageal neoplasm*" OR "oesophageal neoplasm*" OR "gastric cancer" OR "stomach cancer" OR "gastric neoplasm*" OR "stomach neoplasm*")<br>#2 TS=("endoscopic screening" OR "endoscopy screening" OR "screening endoscopy" OR "upper gastrointestinal endoscopy" OR "upper GI endoscopy" OR "screening gastroscopy" OR "screening esophagogastroduodenoscopy" OR (screen* NEAR/5 endoscop*) OR (screen* NEAR/5 gastroscop*) OR (screen* NEAR/5 esophagogastroduodenoscop*) OR (screen* NEAR/5 EGD))<br>#3 TS=("screening uptake" OR "screening participation" OR "screening attendance" OR "screening adherence" OR "screening compliance" OR "participation rate" OR "uptake rate" OR "attendance rate" OR "screening rate" OR "screening behavior" OR "screening behaviour" OR "screening utilization" OR "screening utilisation" OR ((participation OR uptake OR attendance OR adherence OR compliance OR intention OR willingness OR acceptability) NEAR/5 (screen* OR endoscop* OR gastroscop* OR esophagogastroduodenoscop* OR EGD)))<br>#4 TS=(((barrier* OR facilitator* OR obstacle* OR enabler* OR determinant* OR predictor* OR "associated factor*" OR "influencing factor*") NEAR/5 (uptake OR participation OR attendance OR adherence OR compliance OR intention OR willingness OR acceptability OR screen*)) OR ((awareness OR view* OR perspective* OR perception* OR opinion* OR experience* OR challenge*) NEAR/5 (screen* OR endoscop* OR gastroscop* OR esophagogastroduodenoscop* OR EGD)))<br>#5 #3 OR #4<br>#6 #1 AND #2 AND #5

Database	CINAHL with full text (130)
URL	https://research.ebsco.com/c/jixrav/search
Time	Inception to 2026.5
Search strategy	((“upper gastrointestinal cancer*” OR “upper gastrointestinal neoplasm*” OR “upper gastrointestinal tract cancer*” OR “upper digestive tract cancer*” OR “upper digestive tract neoplasm*” OR “esophageal cancer” OR “oesophageal cancer” OR “esophageal neoplasm*” OR “oesophageal neoplasm*” OR “gastric cancer” OR “stomach cancer” OR “gastric neoplasm*” OR “stomach neoplasm*”)) AND ((“endoscopic screening” OR “endoscopy screening” OR “screening endoscopy” OR “upper gastrointestinal endoscopy” OR “upper GI endoscopy” OR “gastroscopy” OR “screening gastroscopy” OR “esophagogastroduodenoscopy” OR “EGD”) AND ("participation" OR "uptake" OR "attendance" OR "adherence" OR "compliance" OR "intention" OR "willingness" OR "acceptability")) AND ((barrier* OR facilitator* OR obstacle* OR challenge* OR enabler* OR determinant* OR predictor* OR factor* OR influence* OR views* OR Awareness OR perspective* OR perception OR Opinion* OR Experience* OR Challenge*))

Database	China National Knowledge Infrastructure (CNKI) (802)
URL	http://www.cnki.net/
Time	Inception to 2026.5
Search strategy	（篇关摘：上消化道癌症 + 上消化道恶性肿瘤 + 消化系统肿瘤 + 上消化道癌 + 食道癌 + 食管肿瘤 + 胃肿瘤 + 胃癌(精确)） AND （篇关摘：内镜 + 上消化道内镜 + 胃镜 + 食管胃十二指肠镜(精确)）AND（篇关摘：筛查率 + 筛查参与 + 筛查行为 + 参与 + 依从性 + 接受性 + 可行性 + 意愿 + 意向 (精确)）OR（篇关摘：影响因素 + 阻碍因素 + 障碍因素 + 促进因素(精确)）

Database	WANFANG DATA (51)
URL	http://www.wanfangdata.com.cn/index.html
Time	Inception to 2026.5
Search strategy	题名或关键词：(上消化道癌症 OR 上消化道恶性肿瘤 OR 消化系统肿瘤 OR 上消化道癌 OR 食道癌 OR 食管肿瘤 OR 胃肿瘤 OR 胃癌) and 题名或关键词:(内镜筛查 OR 上消化道内镜筛查 OR 胃镜筛查 OR 筛查性内镜 OR 上消化道内镜 OR 胃镜检查 OR 食管胃十二指肠镜) and (题名或关键词:(影响因素 OR 阻碍因素 OR 障碍因素 OR 促进因素) OR 题名或关键词:(筛查参与 OR 筛查行为 OR 参与 OR 依从性 OR 接受性 OR 可行性 OR 意愿 OR 意向))

Database	VIP (39)
URL	https://qikan.cqvip.com/
Time	Inception to 2026.5
Search strategy	M = (上消化道癌症 OR 上消化道恶性肿瘤 OR 消化系统肿瘤 OR 上消化道癌 OR 食道癌 OR 食管肿瘤 OR 胃肿瘤 OR 胃癌) AND M = (内镜筛查 OR 上消化道内镜筛查 OR 胃镜筛查 OR 筛查性内镜 OR 上消化道内镜 OR 胃镜检查 OR 食管胃十二指肠镜) AND M = ((影响因素 OR 阻碍因素 OR 障碍因素 OR 促进因素) OR (筛查参与 OR 筛查行为 OR 参与 OR 依从性 OR 接受性 OR 可行性 OR 意愿 OR 意向))

Table S2. Quality assessment of cross-sectional studies using the Agency for Healthcare Research and Quality (AHRQ) checklist (n = 36).

Study	Q1	Q2	Q3	Q4	Q5	Q6	Q7	Q8	Q9	Q10	Q11	Total	Quality
Liu et al., 2008	Y	Y	Y	Y	U	U	U	N	U	Y	U	5	Moderate
Hahm et al., 2011	Y	Y	Y	Y	U	Y	Y	Y	Y	Y	Y	10	High
Lee et al., 2011	Y	Y	Y	Y	U	U	Y	N	U	Y	U	6	Moderate
Choi et al., 2012	Y	Y	Y	Y	U	Y	Y	Y	Y	Y	Y	10	High
Lee et al., 2015	Y	Y	Y	Y	U	U	Y	Y	Y	Y	U	8	High
Yi et al., 2017	Y	Y	Y	Y	U	Y	U	N	U	Y	Y	7	Moderate
Lin et al., 2019	Y	Y	Y	Y	U	Y	U	N	U	Y	U	6	Moderate
Zhang et al., 2019	Y	Y	Y	Y	U	U	U	N	U	Y	Y	6	Moderate
Xiao et al., 2020	Y	Y	Y	Y	U	Y	Y	Y	Y	Y	U	9	High
Cai et al., 2021	Y	Y	Y	Y	U	U	Y	N	U	Y	U	6	Moderate
Li Jun et al., 2021	Y	Y	Y	Y	U	Y	U	N	U	Y	U	6	Moderate
Jiang Li et al., 2021	Y	Y	Y	Y	U	Y	U	N	Y	Y	U	7	Moderate
Xiong et al., 2021	Y	Y	Y	Y	U	U	U	N	U	Y	U	5	Moderate
Li He et al., 2022	Y	Y	Y	Y	U	Y	U	Y	U	Y	U	7	Moderate
Li Qingyu et al., 2022	Y	Y	Y	Y	U	U	U	Y	U	Y	U	6	Moderate
Ryu et al., 2022	Y	Y	Y	Y	U	Y	U	N	U	Y	Y	7	Moderate
Du et al., 2023	Y	Y	Y	Y	U	U	U	N	U	Y	Y	6	Moderate
Guo Lanwei et al., 2023	Y	Y	Y	Y	U	Y	U	N	U	Y	U	6	Moderate
Yang Lei et al., 2023	Y	Y	Y	Y	U	Y	U	Y	U	Y	Y	8	High
Zhu Yongjing et al., 2023	Y	Y	Y	Y	U	U	U	N	U	Y	U	5	Moderate
Du Jia et al., 2024	Y	Y	Y	Y	U	Y	U	Y	U	Y	U	7	Moderate

Study	Q1	Q2	Q3	Q4	Q5	Q6	Q7	Q8	Q9	Q10	Q11	Total	Quality
Wu Qiong et al., 2024	Y	Y	Y	Y	U	Y	U	Y	U	Y	U	7	Moderate
Deng Jiankai et al., 2025	Y	Y	Y	Y	U	U	U	N	U	Y	U	5	Moderate
Li Mengxia et al., 2025	Y	Y	Y	Y	U	U	U	Y	U	Y	U	6	Moderate
Shen Huan et al., 2026	Y	Y	Y	Y	U	U	U	N	U	Y	U	5	Moderate
Fang Zunlong et al., 2026	Y	Y	Y	Y	U	Y	U	N	Y	Y	U	7	Moderate
Kwon et al., 2009	Y	Y	Y	Y	U	N	Y	Y	N	N	U	6	Moderate
Shin et al., 2012	Y	Y	Y	Y	U	Y	Y	Y	Y	Y	U	9	High
Chang et al., 2015	Y	Y	Y	Y	U	Y	Y	Y	Y	U	U	8	High
Guo et al., 2019	Y	Y	Y	Y	U	Y	Y	Y	Y	Y	U	9	High
Jia et al., 2019	Y	Y	Y	Y	U	Y	Y	Y	U	Y	U	8	High
Dong et al., 2022	Y	Y	Y	Y	U	Y	Y	Y	N	N	U	7	Moderate
Zhong et al., 2022	Y	Y	Y	N	U	Y	Y	Y	N	Y	U	7	Moderate
Sheng et al., 2024	Y	Y	Y	N	U	Y	Y	Y	N	Y	U	7	Moderate
Xia et al., 2024	Y	Y	Y	Y	N	Y	Y	Y	N	N	U	7	Moderate
Chen Yong et al., 2024	Y	Y	Y	Y	U	Y	U	N	U	Y	Y	7	Moderate

Note. Q1.Define the source of information (survey, record review); Q2.List inclusion and exclusion criteria for exposed and unexposed subjects (cases and controls) or refer to previous publications; Q3.Indicate time period used for identifying patients; Q4. Indicate whether or not subjects were consecutive if not population-based; Q5.Indicate if evaluators of subjective components of study were masked to other aspects of the status of the participants; Q6.Describe any assessments undertaken for quality assurance purposes (e.g., test/retest of primary outcome measurements); Q7.Explain any patient exclusions from analysis; Q8. Describe how confounding was assessed and/or controlled; Q9. If applicable, explain how missing data were handled in the analysis; Q10.Summarize patient response rates and completeness of data collection; Q11.Clarify what follow-up, if any, was expected and the percentage of patients for which incomplete data or follow-up was obtained. Abbreviations: Y, Yes; N, No; U, Unclear.

Table S3. Quality assessment of cohort studies using the Newcastle–Ottawa scale (NOS) (n = 12).

Study	Selection				Comparability		Outcome			Total	Quality
	Q1	Q2	Q3	Q4	Q5	Q6	Q7	Q8	Q9		
Chen et al., 2009	*	*	*	*	*	/	*	*	*	********	High
Hamashima et al., 2013	*	*	*	/	/	/	*	*	/	*****	Moderate
Hamashima et al., 2015	*	*	*	*	*	/	*	*	/	*******	High
Feng et al., 2020	*	*	*	*	/	/	*	*	*	*******	High
Hao et al., 2020	*	*	*	*	*	/	*	*	*	********	High
Song et al., 2020	*	*	*	*	*	/	*	*	*	********	High
Zhao et al., 2020	*	*	*	*	*	/	*	*	*	********	High
Chen R. et al., 2021	*	*	*	*	*	*	*	*	*	*********	High
Yu et al.,2022	*	*	*	*	*	*	*	*	*	*********	High
Wang et al., 2024	*	*	*	*	*	*	*	*	*	*********	High
Li et al., 2025	*	/	*	/	*	*	*	/	/	*****	Moderate
Yu et al.,2022	*	*	*	*	*	*	*	*	*	*********	High

Note. Q1. Representative of the exposed cohort; Q2. Selection of external control; Q3. Ascertainment of exposure; Q4. Outcome of interest not present at the start of the study; Q5. Main factor; Q6 Additional factor; Q7. Assessment of outcomes; Q8. Sufficient follow-up time; Q9. Adequacy of follow-up.

Table S4. Study-level estimates included in the meta-analysis of associated factors

item	Author(year)	Influencing factors	OR(Adjusted)	95%CI	Reference gruop
1	Kwon et al.,2009	Positive attitude to health check-up	5.26	4.35-6.35	
		Age (yr) 50-59	1.02	0.83-1.25	vs 40-49
		Age (yr) 60-69	0.95	0.74-1.23	
		Age (yr) ≥ 70	0.82	0.59-1.16	
		Household income-Middle tertile	1.08	0.86-1.36	vs Lowest tertile
		Household income-Highest tertile	1.36	1.06-1.73	
		Alcohol consumption (Frequent binge drinker)	0.71	0.53-0.96	vs Non-binge drinker
		Education-Elementary school	1.66	1.21-2.26	vs Uneducated
		Education-Middle or high school	1.38	1.01-1.89	
		Education-University or higher	1.64	1.13-2.37	
		Marriage status-With spouse	1.05	0.87-1.26	vs Without spouse
		Limitation of general activity-Unlimited	0.95	0.76-1.19	vs Limited
		Visual problem-Unlimited	1.03	0.88-1.21	vs Limited
		Ex-smoker	1.08	0.87-1.33	vs Never
		Current smoker	0.86	0.70-1.06	
		Stress severity-Mild	1.06	0.87-1.30	vs None
		Stress severity-Moderate	1.2	0.98-1.40	
		Stress severity-Severe	1.06	0.80-1.41	
2	Shin et al.,2012	Age (years)-50-59	1.41	1.17-1.70	vs 40-49
		Age (years)-60-69	1.49	1.20-1.84	
		Age (years)-≥70	0.81	0.63-1.04	
		Gender (Female)	0.83	0.67-1.04	vs Male
		Education-≥College	1.40	1.09-1.81	vs ≤Elementary school
		Education-Middle-High school	1.21	1.02-1.44	
		Marital status (Not married (single, divorced, widowed))	1.47	1.22-1.75	vs Married
		Monthly household income-2nd quartile	0.97	0.80-1.18	vs 1st quartile
		Monthly household income-3nd quartile	1.08	0.87-1.34	
		Monthly household income-4nd quartile	1.24	0.99-1.55	
		Alcohol consumption(Frequent binge drinker)	0.74	0.55-0.99	vs Non-binge drinker

item	Author(year)	Influencing factors	OR(Adjusted)	95%CI	Reference gruop
		Ex-smoker	1	0.79-1.27	vs Never
		Current smoker	0.75	0.60-0.94	
		Physical activity of moderate intensity-1-4 times/week	1.14	0.97-1.34	vs Never
		Physical activity of moderate intensity-5-7 times/week	1.19	0.99-1.42	
		Stress-Often	0.9	0.76-1.06	vs Rarely
		Self-reported depression-Yes	0.99	0.82-1.20	
		Self-reported health status-Fair	0.93	0.78-1.09	vs Healthy
		Self-reported health status-Unhealthy	1.13	0.96-1.33	
		Dietary habits-Try to eat more vegetables and fruits-Somewhat	0.98	0.84-1.16	vs Very
		Dietary habits-Try to eat more vegetables and fruits-Not at all	1.09	0.89-1.34	
		Dietary habits-Try to have food as bland as possible-Somewhat	1.08	0.93-1.26	vs Very
		Dietary habits-Try to have food as bland as possible-Not at all	0.89	0.75-1.06	
		Dietary habits-Daily sodium intake->2000 mg/day	1.02	0.84-1.24	vs Dietary habits-Daily sodium intake-≤2000 mg/day
3	Chang et al.,2015	50-59	1.48	1.31-1.66	vs 40-49
		60-69	1.68	1.46-1.94	
		≥70	1.05	0.89-1.25	
		Household income-Quartile 4	1.49	1.29-1.72	vs Lower income level
		Household income-Quartile 3	1.17	1.02-1.34	vs Lower income level
		Household income-Quartile 2	1.07	0.94-1.21	vs Lower income level
		Education-≥College	1.34	1.14-1.56	vs Elementary school or less
		Education-High school	1.05	0.92-1.19	
		Education-Middle school	1.13	0.99-1.29	
		Marital status (with spouse)	1.42	1.26-1.60	vs Without spouse

item	Author(year)	Influencing factors	OR(Adjusted)	95%CI	Reference gruop
		Employment-Having a job	0.99	0.90-1.09	vs No job or no activity
		NHI vs. Medicaid	1.16	0.94-1.43	vs NHI
		Private health insurance (Yes)	1.47	1.32-1.64	vs No insurance
		History of upper gastrointestinal system disease (Yes)	1.35	1.15-1.57	vs No history
		Current smoker	0.69	0.62-0.77	Current smoker
		Limitation of activity(Yes)	1.11	1.00-1.24	vs No limitation
4	Lee et al., 2015	Gender(men)	1.08	1.08-1.08	vs Women
		Age, y (40-49)	5.96	5.92-6.00	vs 80+
		(50-59)	5.21	5.18-5.25	
		(60-69)	3.55	3.53-3.58	
		70-79	1.88	1.86-1.89	
		Socioeconomic status (national health insurance (high))	2.24	2.23-2.25	vs MAP
		Socioeconomic status (national health insurance (low))	1.72	1.71-1.72	
5	Guo et al.,2019	Female	1.24		vs Male
		Education-≥College	1.36	1.23-1.51	vs Primary school or below
		Education-Junior/Senior high school	1.17	1.08-1.27	
		Married	1.28	1.15-1.43	vs Not married
		Former Smoking	1.24	1.11-1.39	vs Not smoking
		Current smoker	1.11	1.02-1.20	vs Not smoking
		Current drinking	1.15	1.07-1.23	vs Not drinking
		Former drinking	0.97	0.86-1.10	
		Physical activity <3 times/wk	1.22	1.16-1.29	≥3 times/wk
		History of upper gastrointestinal system disease(Yes)	1.88	1.74-2.02	vs No history
		Family history of UGC (Yes)	1.60	1.51-1.68	vs No family history
		45-49	1.15	1.04-1.27	vs 40-44
		50-54	1.19	1.08-1.31	

item	Author(year)	Influencing factors	OR(Adjusted)	95%CI	Reference gruop
		55-59	1.20	1.08-1.33	
		60-64	1.20	1.08-1.32	
		70-74	0.75	0.63-0.90	
		65-69	1.05	0.94-1.17	
6	Jia et al.,2019	Higher cancer prevention knowledge levels-Q4 (>70)	1.511	1.398−1.632	vs ＜40
		Q3 (56−70)	1.225	1.138−1.319	
		Q2 (40−55)	1.086	1.012−1.165	
7	Dong et al.,2022	Gender-Famale	1.27	1.22-1.32	vs Men
		50-59	1.29	1.24-1.34	vs 40-49
		60-69	1.37	1.32-1.43	
		Education-≥College	1.66	1.46-1.90	vs Elementary school or less
		Education-Junior/Senior high school	1.01	0.97-1.05	
		divorced	1.43	1.27-1.62	vs Married
		widowed	1.12	1.03～1.23	
		Not married	0.86	(0.72-1.03)	
		Household income-Q1-＞50 000	1.24	1.16～1.31	vs 0～1 0000
		Household income-Quartile 3 ＞30 000～50 000	1.43	1.36-1.50	
		Household income-Quartile 2 ＞10 000～30 000	1.46	1.41-1.52	
		Body mass index (BMI)-underweight	0.85	0.73-0.98	vs Normal
		Body mass index (BMI)-Overweight	0.96	0.93-0.99	
		Body mass index (BMI)-Obese / Obesity	0.97	0.91-1.03	
		Drinking untreated water sources (shallow water)	1.33	1.27～1.38	vs tap water (treated)
		Drinking untreated water sources (deep well water)	1.31	1.24～1.37	
		No smoking	1.27	1.21～1.33	vs smoking
		No drinking	1.17	1.11～1.22	vs drinking
		History of upper gastrointestinal system diseases -1 condition	1.32	1.27～1.37	vs no history
		History of upper gastrointestinal system diseases -2	1.93	1.81～2.06	

item	Author(year)	Influencing factors	OR(Adjusted)	95%CI	Reference gruop
		conditions			
		History of upper gastrointestinal system diseases -3 conditions	2.47	2.17～2.81	
		Family history of UGC (Yes)	1.27	1.17～1.37	vs no family history
8	Li Q et al., 2022	Age（50-59）	1.2	1.02-1.4	vs 40-49
		≥60	1.46	1.25-1.71	vs 40-49
		Education（high school/technical secondary school）	1.21	1.07-1.36	vs Junior high school or below
		College degree or above	1.21	1.07-1.38	vs Junior high school or below
		Psychological trauma or depression（Yes）	1.15	1.04-1.27	vs no
		History of upper gastrointestinal disease（Yes）	2.19	1.89-2.54-	vs no
		Family history of cancer（Yes）	1.82	1.63-2.02	vs no
		Current smoking status（Smoker）	0.88	0.79-0.98	vs no
		Regular physical exercise（Yes）	0.88	0.8-0.98	vs no
		Temperature preference of daily diet（Very hot）	1.14	1.03-1.27	vs Cool/moderate
		Household cooking fumes（Slight fumes）	1.32	1.02-1.71	vs no fume
		Household cooking fumes（More fumes）	1.43	1.1-1.87	vs no fume
9	Li H et al., 2022	Gender (Female) [Rural area]	1.52	1.39-1.66	vs men
		Age (50-59 years)	1.38	1.26-1.5	vs 40-49
		60-69 years	1.66	1.51-1.83	vs 40-49
		Education (primary or middle school)	1.15	1.04-1.29	vs uneducated
		High school or above	1.62	1.01-2.6	vs uneducated
		Income (lower-middle income)	1.29	1.17-1.43	vs lower income level
		Upper-middle income	1.28	1.15-1.43	vs lower income level
		High income	1.15	1.04-1.27	vs lower income level
		History of smoking (Yes)	0.86	0.78-0.95	vs None
		Family history of UGC(Yes)	1.16	1.06-1.26	vs None
		History of gastroduodenal ulcer (Yes)	1.42	1.26-1.6	vs None
		History of reflux esophagitis (Yes)	1.26	1.08-1.47	vs None

item	Author(year)	Influencing factors	OR(Adjusted)	95%CI	Reference gruop
		History of superficial gastritis (Yes)	1.35	1.25-1.45	vs None
		Age (50-59 years) [Urban area]	0.93	0.84-1.02	vs 40-49
		60-69 years	0.73	0.66-0.8	vs 40-49
		Education (primary or middle school)	0.55	0.37-0.81	vs Uneducated
		High school or above	0.6	0.4-0.89	vs uneducated
		Per capita annual income (lower-middle income)	0.75	0.67-0.83	vs lower income level
		Upper-middle income	0.73	0.65-0.82	vs lower income level
		High income	0.78	0.7-0.87	vs lower income level
		History of smoking (Yes)	0.85	0.78-0.94	vs No
		Family history of UGC(Yes)	1.42	1.26-1.61	vs No
		History of reflux esophagitis (Yes)	1.34	1.18-1.52	vs No
		History of superficial gastritis (Yes)	1.22	1.13-1.32	vs No
10	Yu et al.,2022	Age (years)-40-44	1.49	1.04 -2.13	vs 69-74
		Age (years)-45-49	1.81	1.37-2.38	
		Age (years)-49-54	1.54	1.17-2.02	
		Age (years)-55-59	1.78	1.37-2.30	
		Age (years)-60-64	1.70	1.32-2.20	
		Age (years)-65-69	1.43	1.09-1.86	
		Female	1.24	1.11-1.38	vs men
		Education-≥College	1.45	1.04-2.02	vs lower educational level
		Education-Senior high school	1.14	0.83-1.56	
		Second smoking (Yes)	1.17	1.04-1.32	vs no second smoking
		History of trauma (Yes)	1.29	1.11-1.50	vs no trauma
		History of mental depression (Yes)	1.39	1.19-1.62	vs no mental depression
		History of upper gastrointestinal system disease (Yes)	1.77	1.52-2.07	vs no history
		Family history of UGC (Yes)	1.37	1.23-1.52	vs no family history
11	Zhong et al.,2022	Educational level	4.994	1.004-24.836	vs lower education level
		Physical exercise 1-3 times/week	2.798	1.275-6.143	vs ＜1 time/week
		Physical exercise ＞3times/week	3.963	1.613-9.735	

item	Author(year)	Influencing factors	OR(Adjusted)	95%CI	Reference gruop
		Alarm symptoms	7.65	3.366-16.154	vs no
		cognitive level	1.222	1.045-1.430	Continuious variable
		Perceived susceptibility	1.435	1.203-1.710	
		Perceived benefits	3.418	2.593-4.506	
		Perceived barriers	0.705	0.642-0.773	
		Subjective norms	1.152	1.021-1.300	
12	Du et al.,2023	Age, years (45-49)	1.06	0.96-1.17	vs 40-44
		50-54	1.13	1.03-1.24	vs 40-44
		55-59	1.11	1.01-1.22	vs 40-44
		60-64	1.05	0.95-1.15	vs 40-44
		65-69	0.94	0.85-1.04	vs 40-44
		70-74	0.82	0.72-0.93	vs 40-44
		Education level (high school/technical secondary school/vocational school)	1.26	1.2-1.33	vs junior high school
		College degree or above	1.6	1.49-1.71	
		Occupation (farmer)	1.11	1.03-1.2	vs technical worker
		Worker	0.9	0.85-0.96	
		Other	1.04	0.97-1.12	
		Fresh vegetable intake (sometimes)	1.14	1.03-1.25	vs few or never
		Often	1.42	1.28-1.58	
		Fresh fruit intake (sometimes)	1.05	0.96-1.14	
		Often	0.83	0.76-0.92	
		Meat intake (sometimes)	1.11	1.02-1.2	
		Often	0.99	0.91-1.07	
		Taste preference (moderate)	0.87	0.82-0.93	vs Salt
		Light/bland	1.01	0.92-1.1	vs Salt
		Fat intake (sometimes)	0.81	0.74-0.87	vs few or never
		Often	0.73	0.67-0.8	vs few or never
		Consumption of scalding-hot foods (Yes)	1.24	1.18-1.31	vs No
		Exposure to cooking oil fumes (relatively high)	1.12	1.06-1.17	vs few

item	Author(year)	Influencing factors	OR(Adjusted)	95%CI	Reference gruop
		Smoking (current smoker)	0.92	0.87-0.96	vs Never
		Former smoker	1.21	1.1-1.33	vs Never
		History of upper gastrointestinal disease (Yes)	1.83	1.73-1.93	vs No
		Hypertension (Yes)	0.84	0.8-0.89	vs No
		Hyperlipidemia (Yes)	1.07	1.01-1.13	vs No
		Diabetes mellitus (Yes)	0.81	0.75-0.86	vs No
		Family history of cancer (Yes)	1.33	1.24-1.41	vs No
		Family history of UGC(Yes)	1.28	1.12-1.46	vs No
		First-degree relatives	1.38	1.23-1.55	vs No
		Family history of esophageal cancer (Yes)	1.21	1.12-1.32	vs No
		Family history of gastric cancer (Yes)	1.26	1.14-1.39	vs No
13	Du et al.,2024	Gender（Female）	1.01	0.949-1.075	vs male
		Age（45-49）	1.123	1.017-1.239	vs 40-44
		50-54	1.114	1.012-1.226	
		55-59	1.173	1.063-1.295	
		60-64	1.119	1.017-1.231	
		65-69	1.03	0.93-1.141	
		70-74	0.725	0.613-0.857	
		BMI（Normal）	0.999	0.879-1.136	vs Thin
		Overweight/obesity	1.031	0.905-1.174	
		Education（High school/vocational school/technical school）	1.126	1.065-1.19	vs Junior high school or below
		College degree or above	1.681	1.57-1.801	
		Marital status（Married）	1.286	0.997-1.659	vs Unmarried
		Divorce/widow	1.368	1.046-1.789	
		Occupational exposure to hazardous substances（Yes）	1.116	1.059-1.175	vs No
		Dietary temperature（Scald）	1.147	1.086-1.212	vs Cool/moderate
		Dietary taste（Heavy salt）	0.993	0.932-1.059	vs Light/moderate

item	Author(year)	Influencing factors	OR(Adjusted)	95%CI	Reference gruop
		Dietary grease（Higher）	1.085	1.018-1.157	vs Lower/moderate
		Pickled and sun dried food（Often）	1.084	1.025-1.146	vs Never/sometimes
		Household cooking fumes（More/many）	1.186	1.122-1.255	vs Smokeless/slightly
		Smoking（Currently smoking）	0.891	0.837-0.949	vs Never
		Stopped smoking	1.041	0.928-1.167	
		Drinking（Currently drinking）	1.053	0.991-1.119	vs Never drink
		Stopped drinking	1.05	0.956-1.154	
		Regular physical exercise（Yes）	0.882	0.836-0.93	vs No
		Mental trauma or depression（Yes）	1.298	1.233-1.368	vs No
		History of upper gastrointestinal diseases（Yes）	2.366	2.212-2.53	vs No
		Family history of UGC(Yes)	1.601	1.525-1.68	vs No
14	Sheng et al.,2024	Education ≤High school	6.316	4.222-9.446	vs college and more
		Family history of cancer (No)	1.875	1.088-3.231	Family history of cancer: yes
		History of upper gastrointestinal system disease (No)	1.707	1.079-2.700	History of digestive system diseases: yes
		lower income level＜3000 CNY	3.019	2.056-4.434	Monthly household income per capita: ≥3000 CNY
		Alarm symptoms(No)	3.605	1.872-6.943	Presence of gastric cancer warning symptoms: yes
		Physical activity <3 times/wk	1.938	1.350-2.782	Physical exercise frequency: ≥3 times/week
		Internal opposition(Yes)	6.589	4.395-9.878	Internal resistance: no
15	Wu et al.,2024	Education level (high school or technical secondary school)	1.194	0.706-2.02	vs Junior high school or below
		College degree or above	3.371	1.793-6.338	vs Junior high school or below
		History of alcohol drinking (Yes)	2.885	1.702-4.888	vs No
		Per capita monthly household income, CNY (2,000-)	1.245	0.791-1.961	vs <2 000
		≥4,000	2.209	1.263-3.861	vs <2 000

item	Author(year)	Influencing factors	OR(Adjusted)	95%CI	Reference gruop
		History of gastrointestinal disease (Yes)	3.007	1.942-4.656	vs No
		Helicobacter pylori infection (Yes)	3.319	1.781-6.184	vs No
		Family history of GC(Yes)	2.036	1.265-3.277	vs No
		Family history of other cancers (Yes)	4.862	2.006-11.783	vs No
		History of gastroscopy (Yes)	1.822	1.028-3.228	vs No
16	Xia et al.,2024	Female	1.160	1.072-1.254	vs men
		45～49	1.151	1.048-1.265	vs 40-44
		50～54	1.184	1.080-1.298	vs 40-44
		55～59	1.197	1.092-1.313	vs 40-44
		60～64	1.198	1.082-1.327	vs 40-44
		65-69	1.029	0.919-1.029	vs 40-44
		70-75	0.683	0.576-0.810	vs 40-44
		Education-≥College	1.363	1.236-1.504	Vs Primary school or below
		junior high/high school	1.138	1.056-1.226	
		unmarried/divorced/wisdowed	1.294	1.151-1.456	vs married
		formor smoking	1.221	1.086-1.374	vs no smoking
		Current smoker	1.030	0.955-1.112	
		former drinker	0.970	0.864-1.089	vs no drinking
		Current drinking	1.115	1.039-1.197	
		Family history of UGC (Yes)	1.402	1.322-1.487	vs no history
		History of reflux esophagitis: yes	1.230	1.137-1.330	vs no history
		History of superficial gastritis: yes	1.281	1.208-1.359	vs no history
		History of gastric ulcer: yes	1.186	1.121-1.256	vs no history
		History of duodenal ulcer: yes	1.186	1.121-1.256	vs no history
		History of gastric polyps: yes	1.343	1.271-1.419	vs no history
17	Li M et al.,2025	Gender (Female)	1.24	1.21-1.27	vs Men
		Age group, years (50-59)	1.41	1.38-1.45	vs 40-49
		≥60	1.74	1.69-1.78	vs 40-49
		BMI classification (Underweight)	1.06	0.98-1.16	vs Normal
		Overweight	0.94	0.92-0.96	vs Normal

item	Author(year)	Influencing factors	OR(Adjusted)	95%CI	Reference gruop
		Obesity	0.91	0.88-0.94	vs Normal
		Smoking (Yes)	0.7	0.68-0.71	vs No
		Marital status (Unmarried)	1	0.9-1.12	vs Married
		Divorced or widowed	1.06	1.02-1.1	vs Married
		Annual household income, CNY 10,000 (1-<3)	1.19	1.14-1.24	vs <1
		3-<6	1.34	1.29-1.4	vs <1
		6-<10	1.59	1.52-1.66	vs <1
		≥10	2.15	2.03-2.28	vs <1
		Drinking water source (cellar water, pond water, or shallow well water)	1.3	1.2-1.41	vs tap water (treated)
		Lake water or river water	1.3	1.01-1.67	vs tap water (treated)
		Deep well water or spring water	1.13	1.1-1.15	vs tap water (treated)
		Family history of cancer (Yes)	1.11	1.08-1.13	vs No
18	Li B et al.,2025	Age group（50-54）	1.04	0.98-1.11	vs 45-49
		55-59	1.05	0.98-1.12	vs 45-49
		60-64	0.95	0.88-1.02	vs 45-49
		65-69	0.8	0.74-0.87	vs 45-49
		70-74	0.6	0.53-0.68	vs 45-49
		Education（Low）	0.65	0.6-0.7	vs High
		Medium	0.75	0.7-0.8	vs High
		Occupational exposure to hazards（Yes）	1.19	1.14-1.25	vs No
		Hot foods（Yes）	1.27	1.21-1.33	vs No
		Physical exercise（≥3 times/week）	0.88	0.84-0.93	vs <3 times/week
		History of upper gastrointestinal disease（Yes）	2.37	2.23-2.52	vs No
		Number of baseline comorbidity（1）	1.08	1.02-1.15	vs 0
		2	1.23	1.15-1.32	vs 0
		≥3	1.24	1.16-1.33	vs 0
		Family history of UGIC（First-degree relatives）	1.63	1.55-1.72	vs No
		Second-degree relatives	1.62	1.47-1.78	vs No

item	Author(year)	Influencing factors	OR(Adjusted)	95%CI	Reference gruop
		Third-degree relatives	1.38	1.1-1.72	vs No
	Li B et al.,2025	Economic status（Developing areas）	0.69	0.66-0.73	vs Developed areas
		Distance to screening hospital（≥5 km）	0.87	0.83-0.92	vs <5 km
		Number of media（>3）	1.17	1.11-1.24	vs ≤3
		Number of mobilization methods（>3）	1.08	1.03-1.14	vs ≤3
		Allowance（Yes）	1.49	1.41-1.58	vs No
		Screening green channel（Yes）	1.06	1.00-1.13	vs No
19	Song et al.,2025	Education-high	1.10	1.03-1.17	vs. low
		Education-Intermediate	1.10	1.03-1.17	
		50-59 years	1.05	0.99-1.11	vs 40-49
		60-69 years	1.03	0.97-1.10	
		Female	0.97	0.93-1.02	vs men
		Married	1.05	0.94-1.16	vs Unmarried/ divorced/widowed
		History of upper gastrointestinal diseases	2.30	2.17-2.44	vs no
		Family history of cancer (Yes)	2.00	1.90-2.11	vs no

Note: OR, odds ratio; CI, confidence interval. Only adjusted odds ratios from multivariable models were extracted and pooled. For education level, categories were harmonized as higher education versus lower education. When necessary, ORs and 95% CIs were inverted to ensure that an OR > 1 consistently indicated a higher likelihood of endoscopic screening uptake among individuals with higher education. Studies with non-comparable education categories or inconsistent reference groups were not pooled and were summarized narratively.

Figure S1.Sensitivity analysis for meta-analyses of pooled participation rates

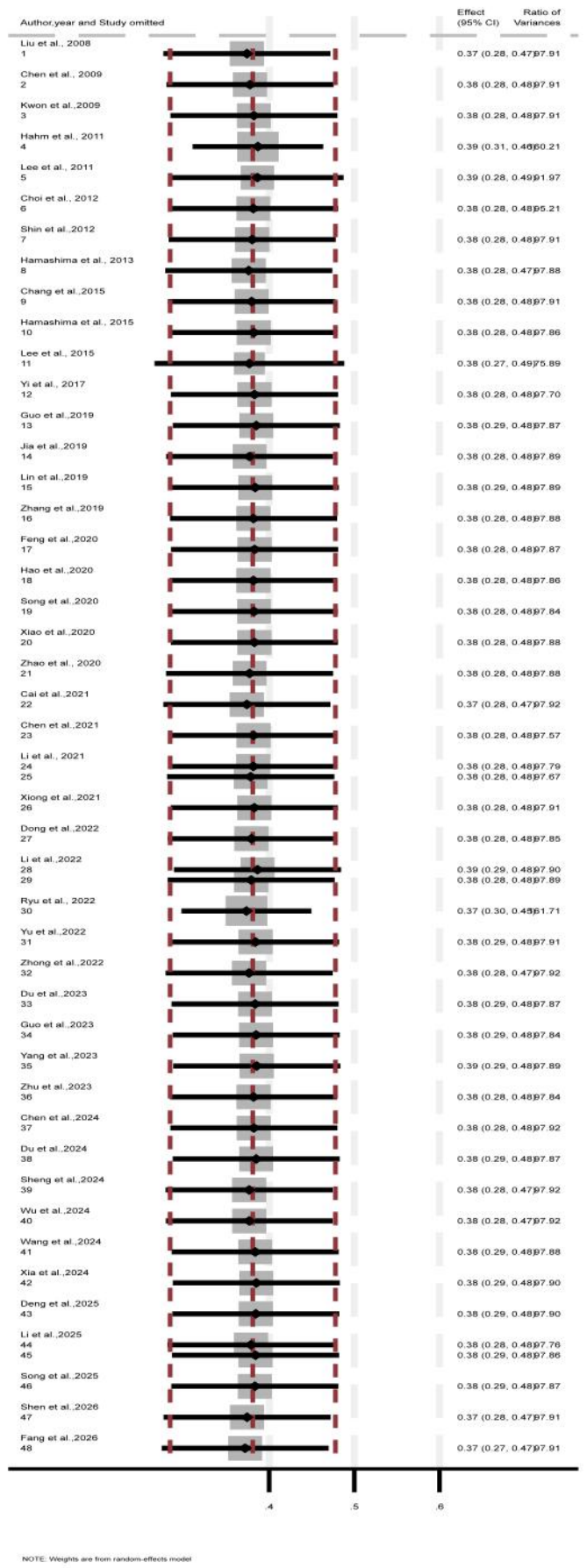

Figure S2. Forest plots of subgroup analyses for upper gastrointestinal endoscopic screening uptake.

a) Country

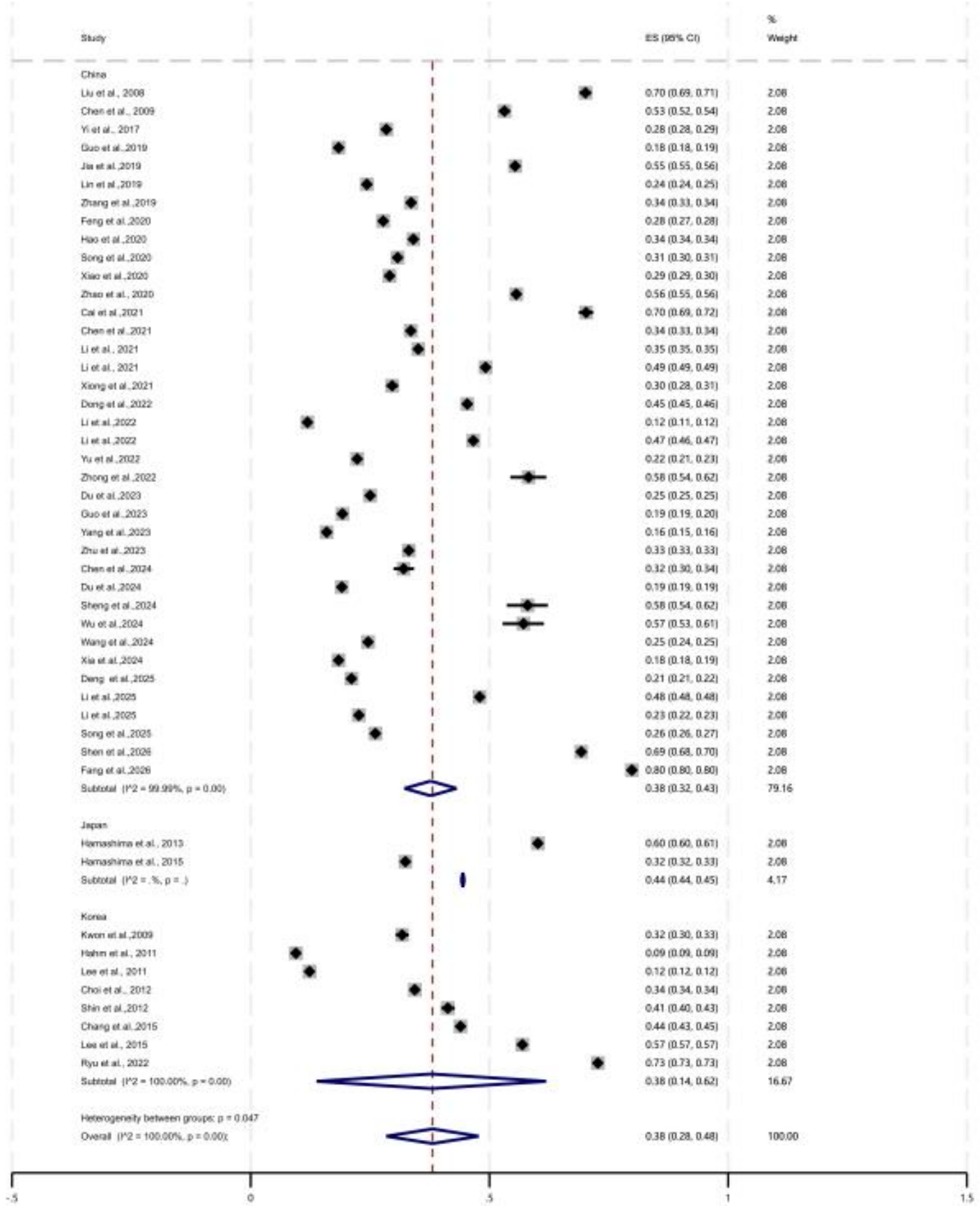

b) Outcome assessment

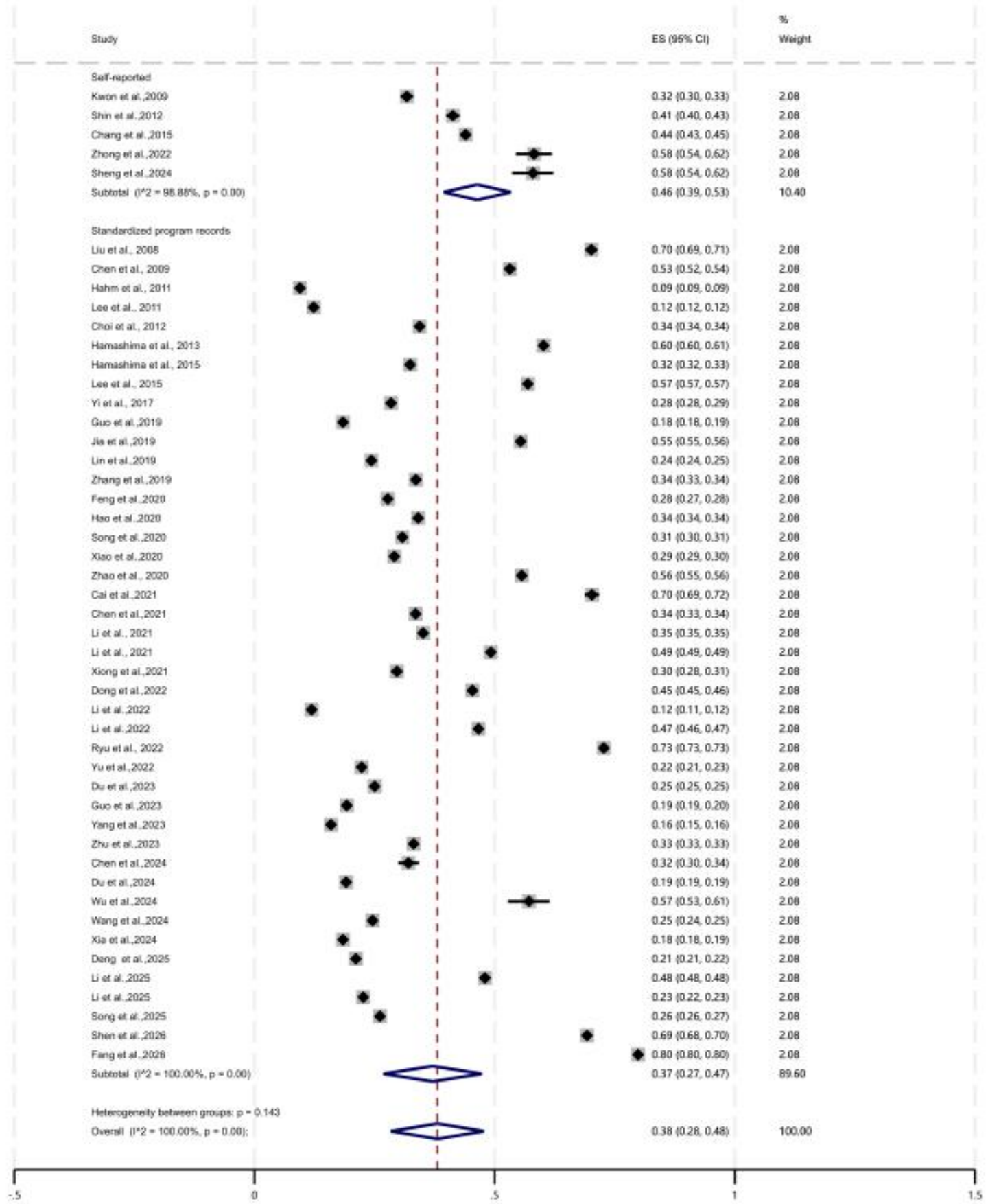

c) Study design

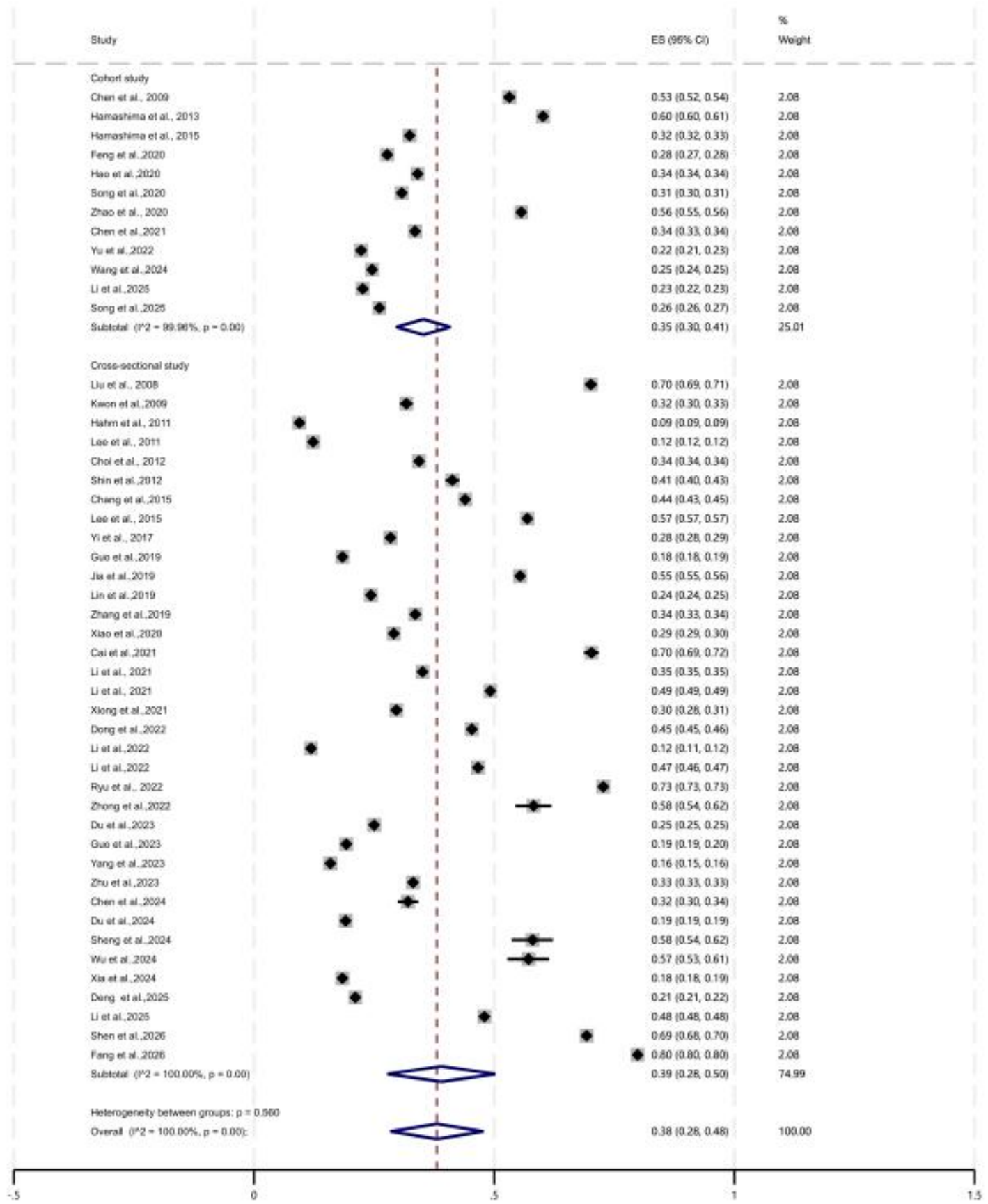

d) Study quality

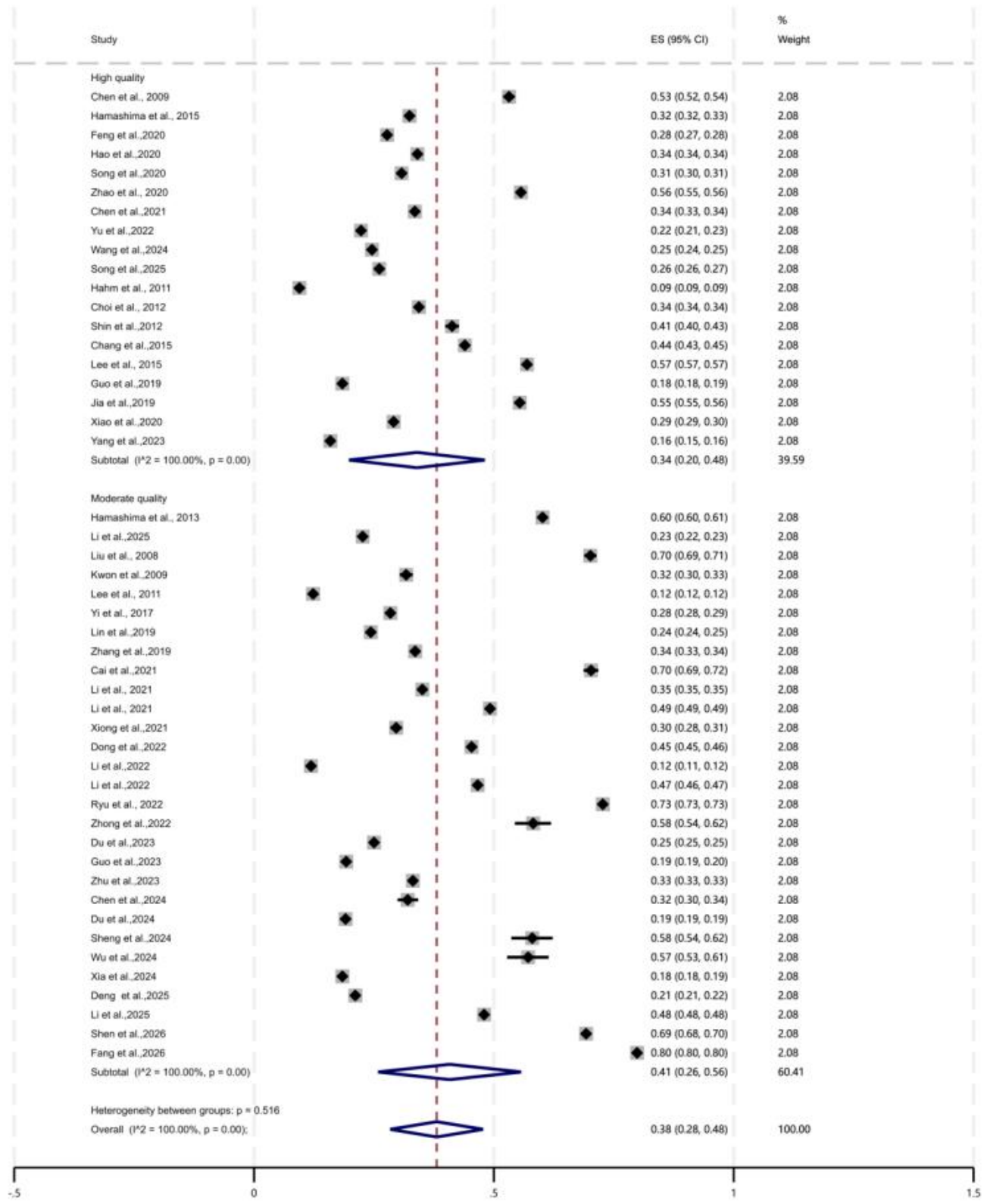

e) Sample Scale

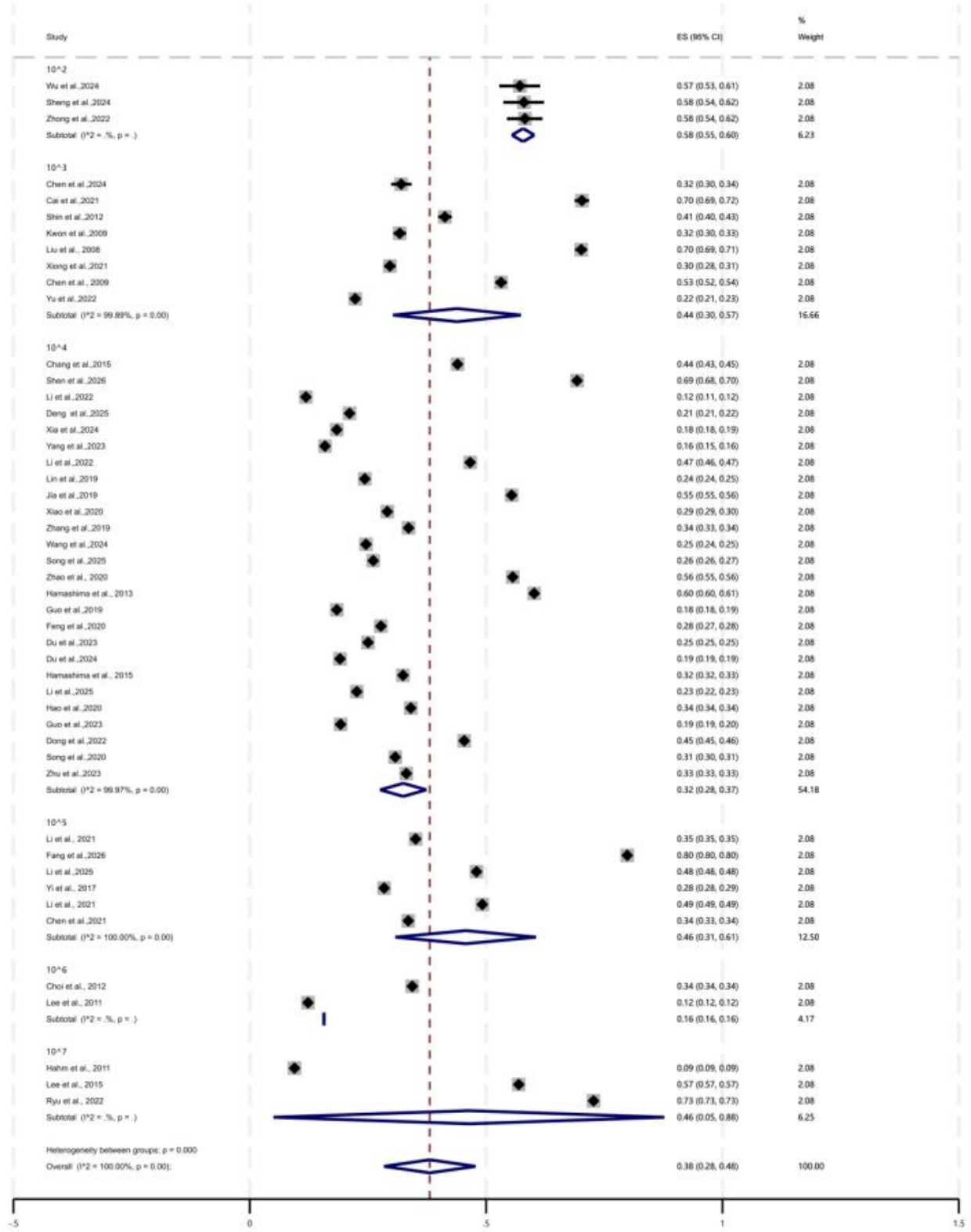

f) Population

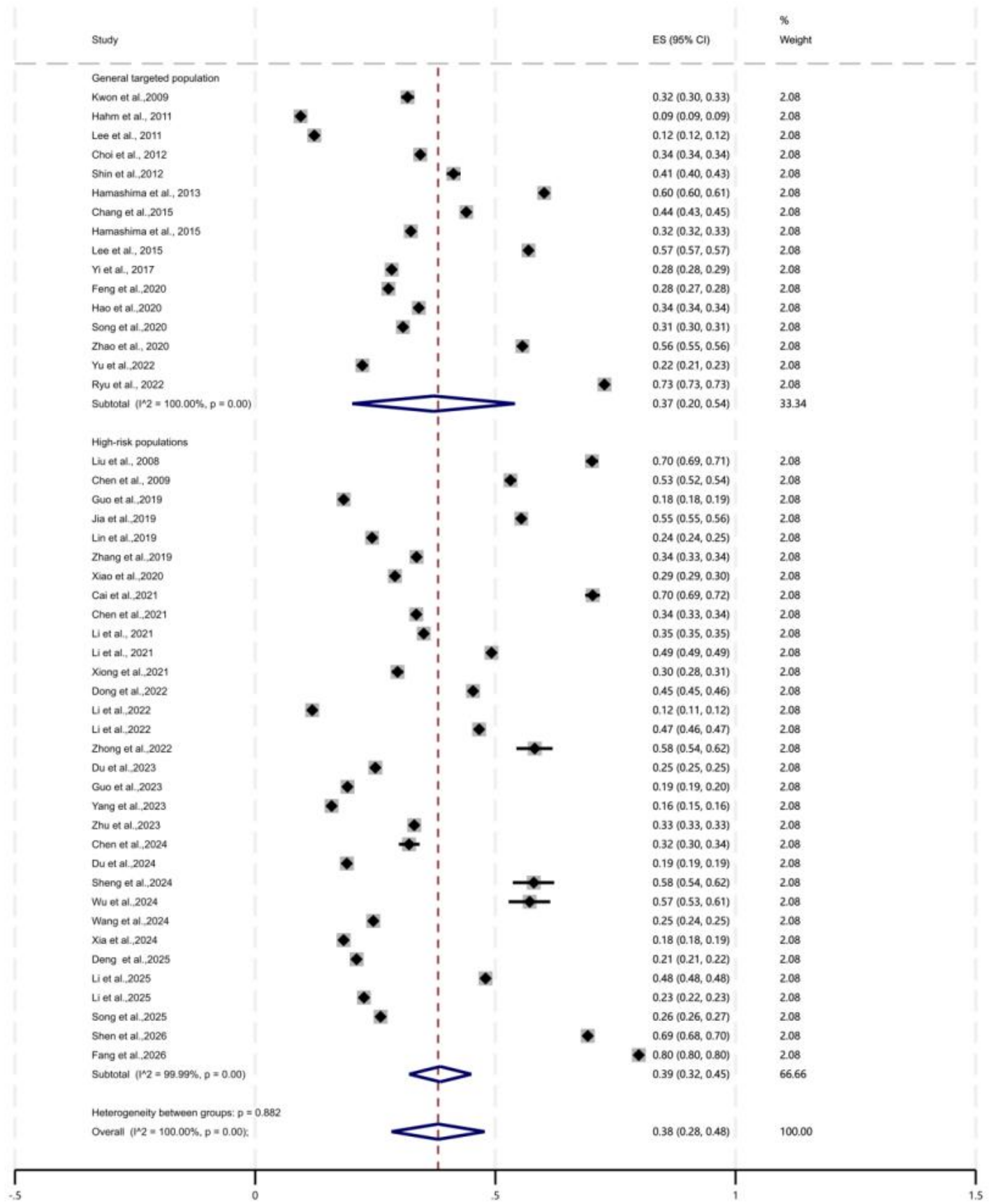

g)Data collection period

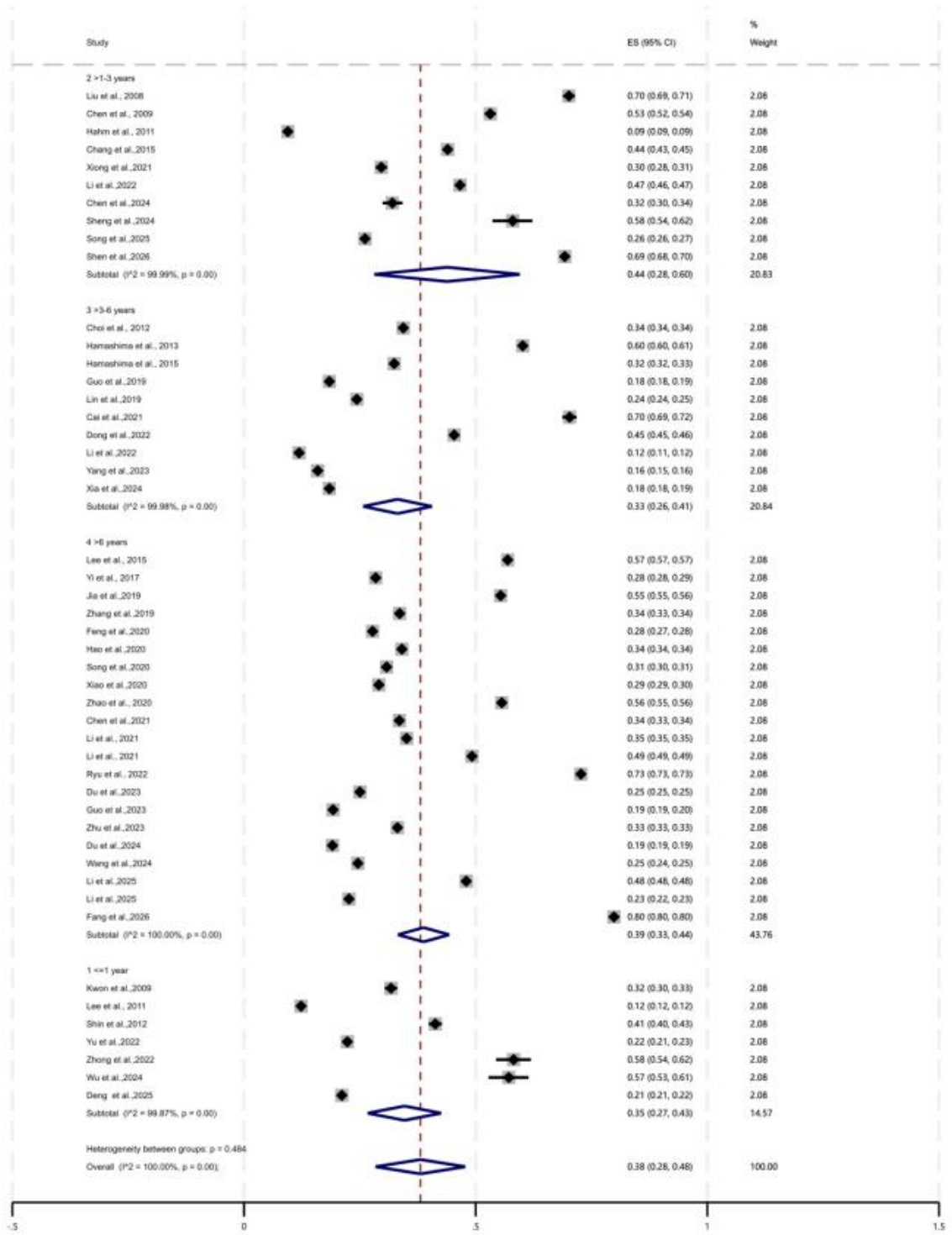

h) Government-led program (Yes/No)

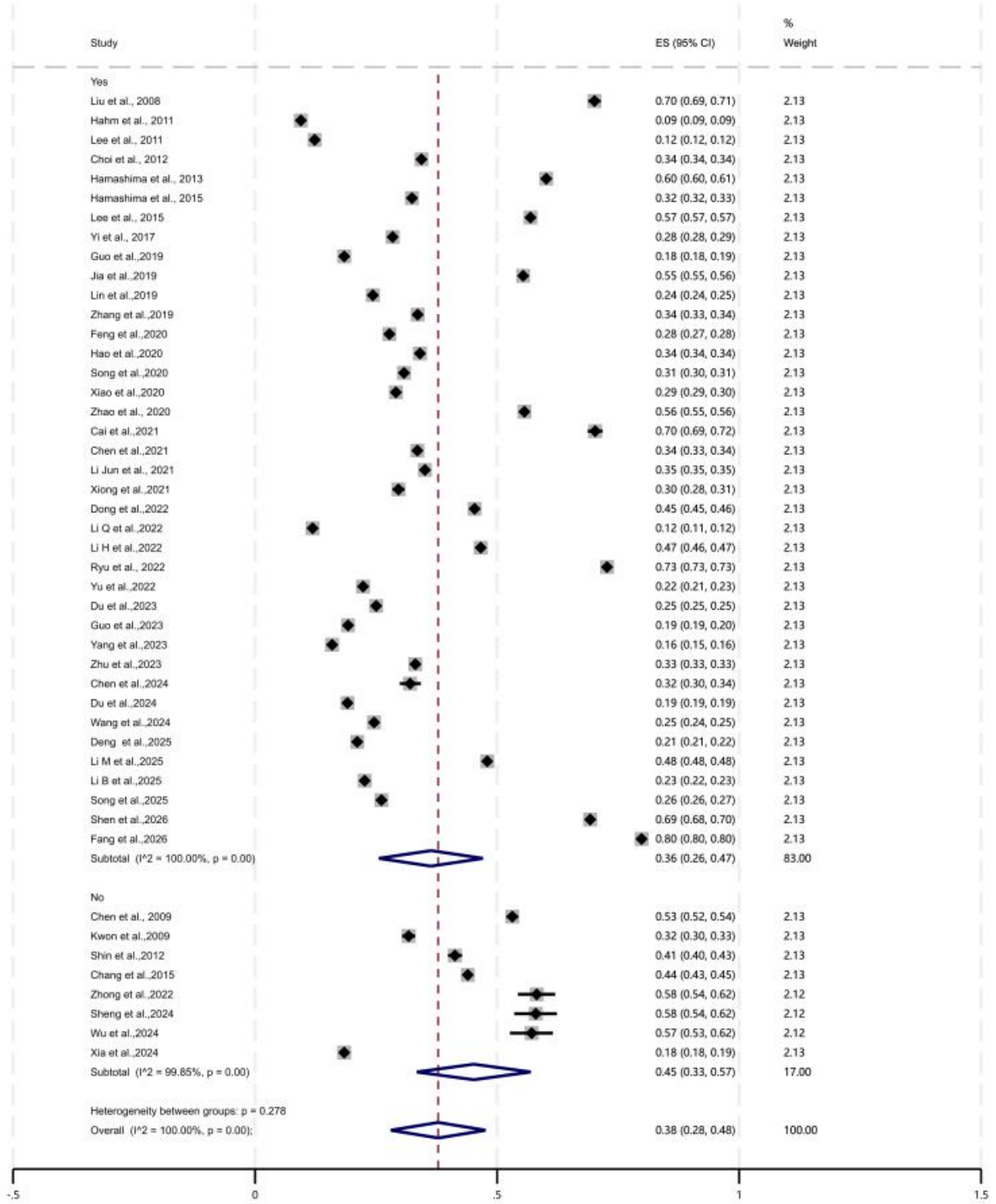

i) Two-step strategies or not

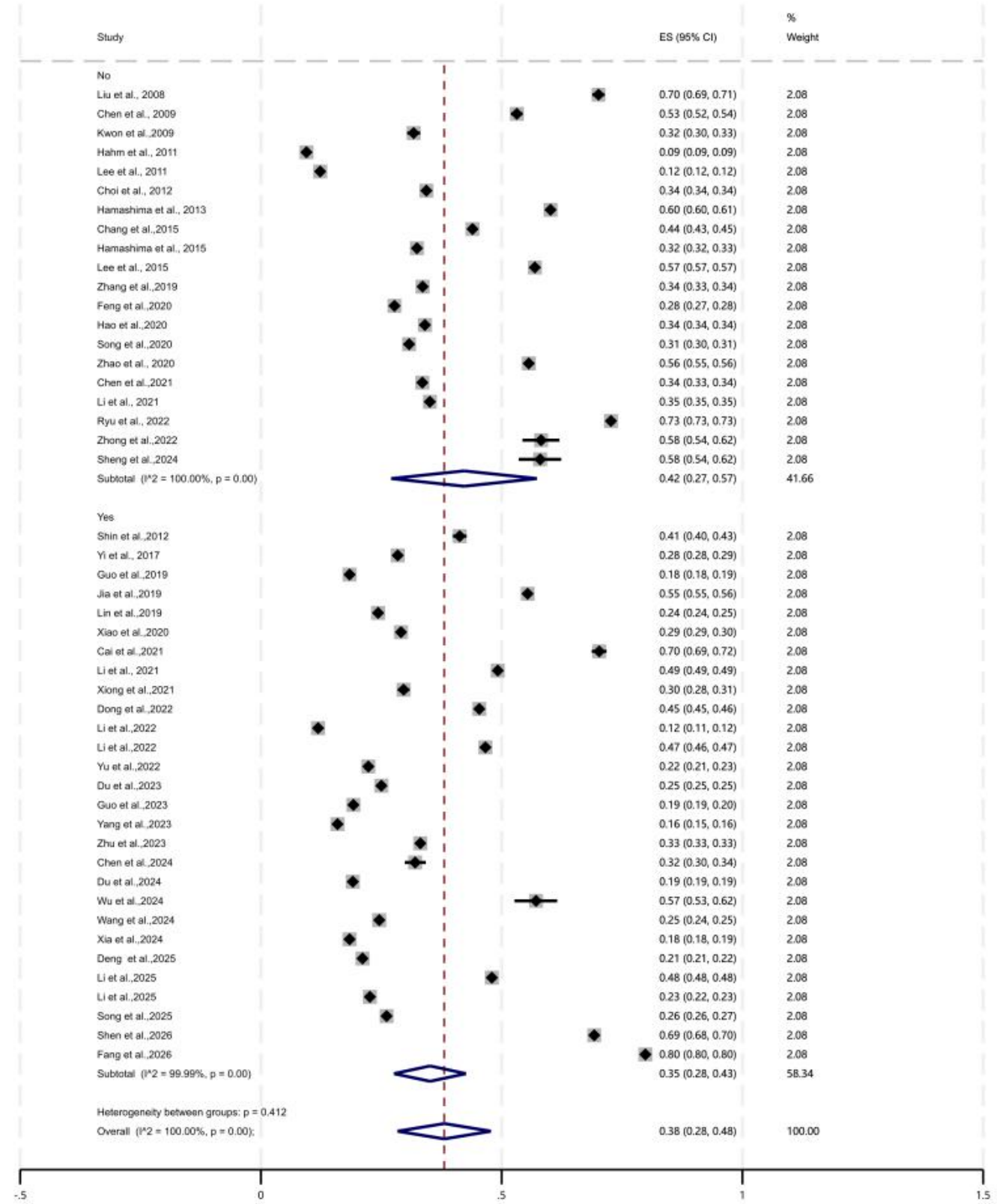

Figure S3. Forest plots of factors associated with upper gastrointestinal endoscopic screening uptake

a. Gender (Female vs Male, n = 10)

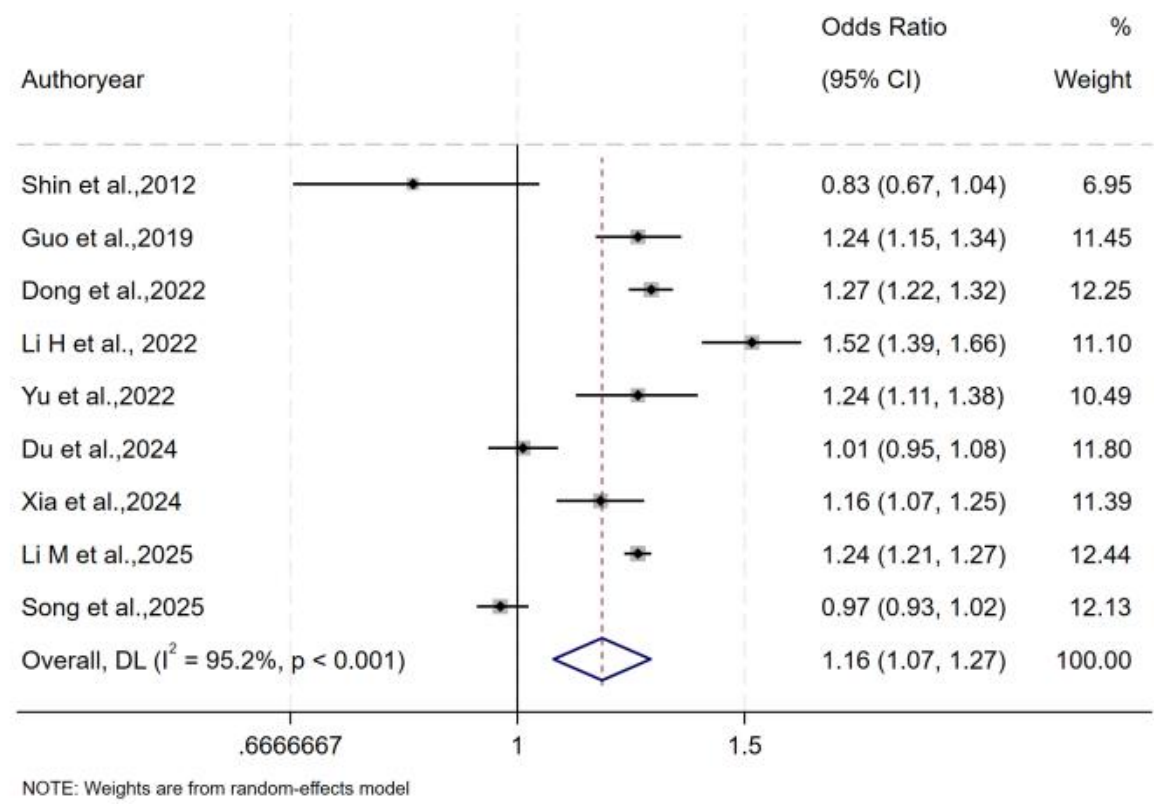

*Lee et al.,2015 reported OR = 0.93 with 95% CI = 0.93-0.93. This estimate was not included in the pooled analysis because the standard error could not be derived from an interval with identical lower and upper limits. The study was retained in the extraction table.

b. Family history of UGC

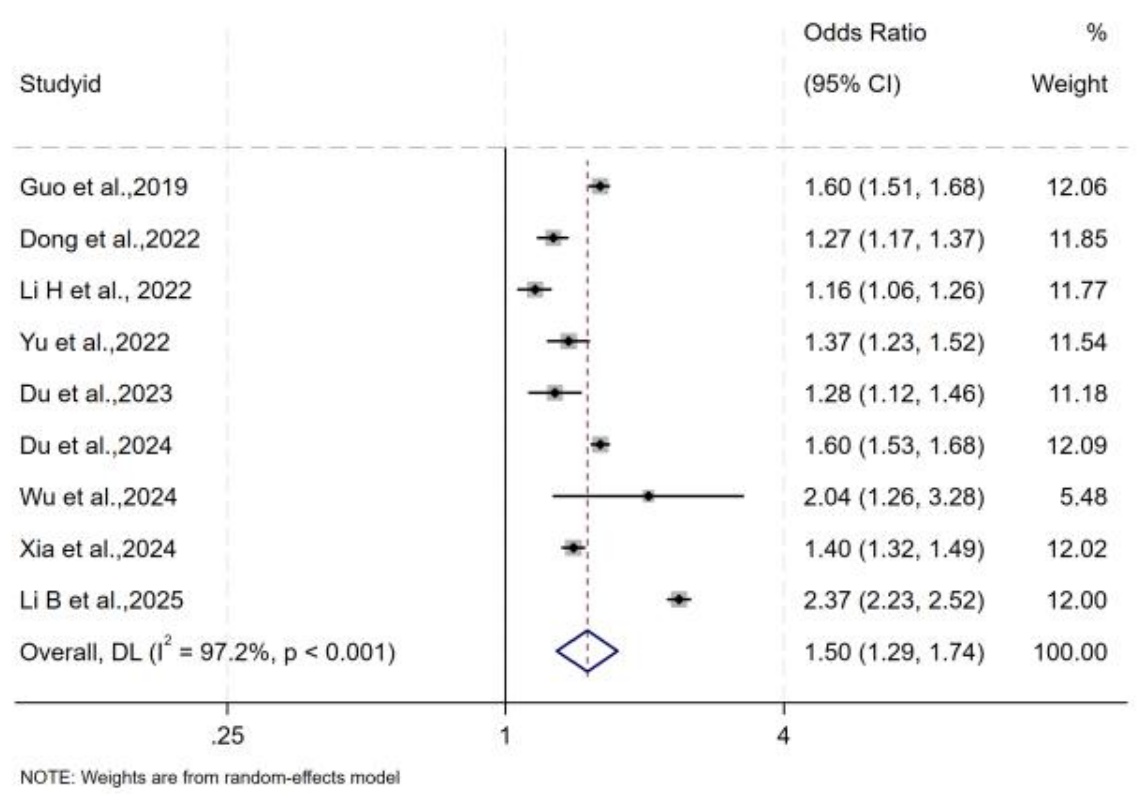

c. History of upper gastrointestinal system diseases

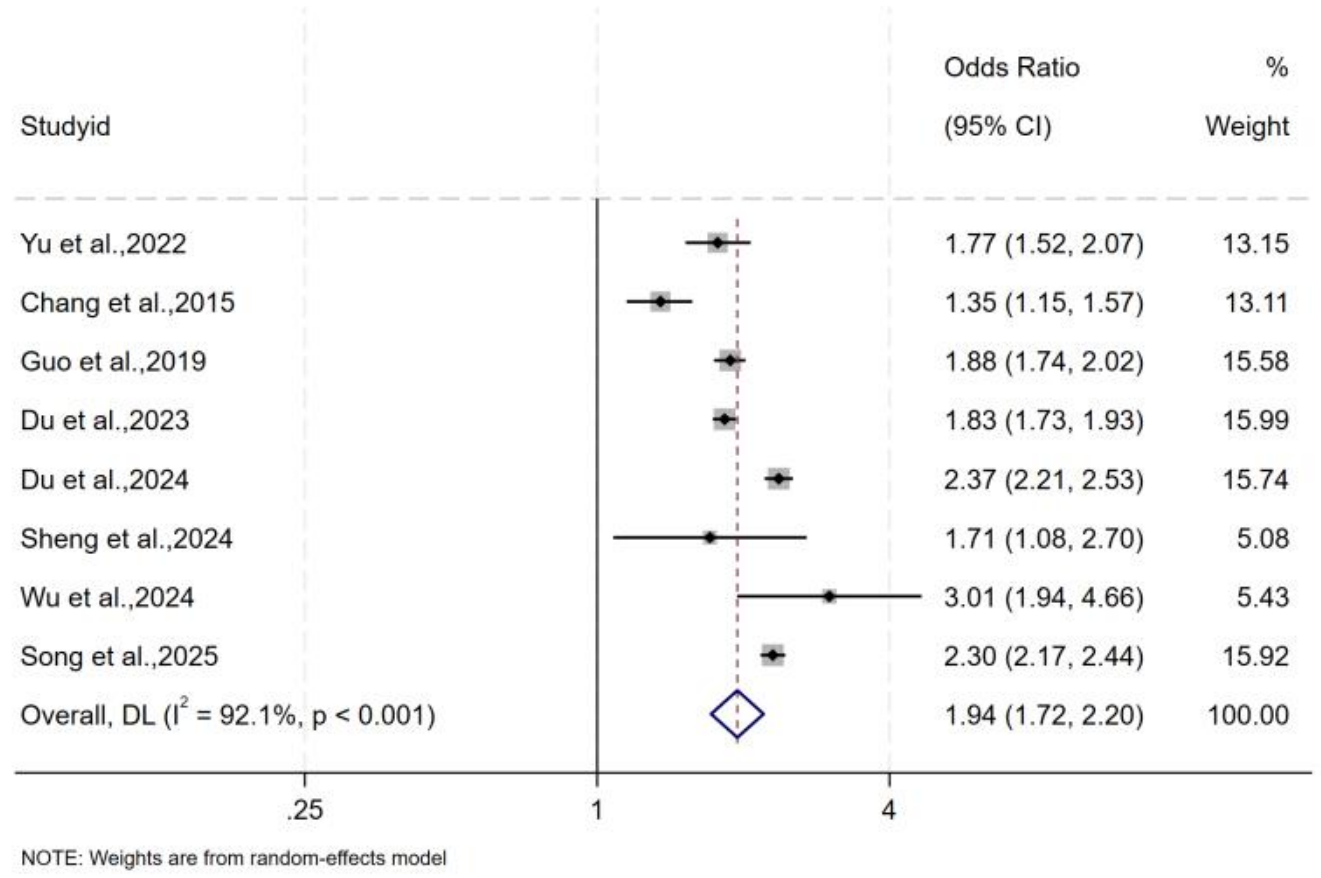

d. Family history of cancer

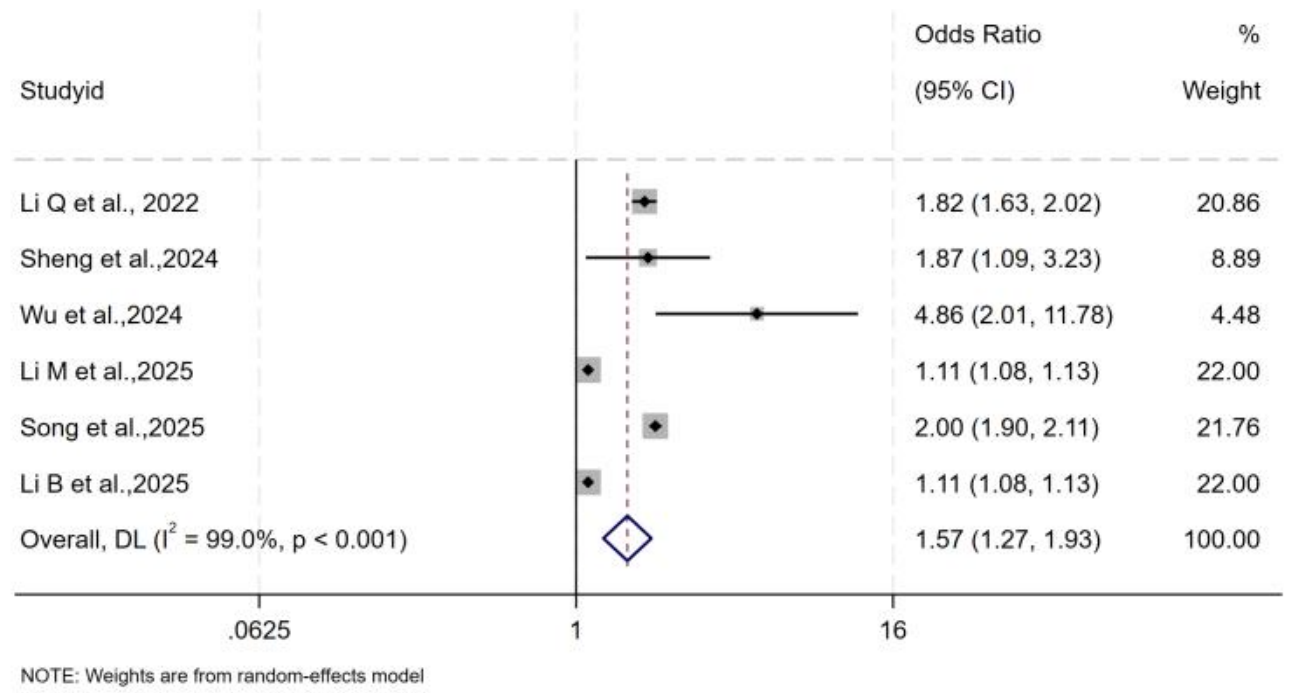

e. Marital status (Married/with spouse vs unmarried/divorced/widowed)

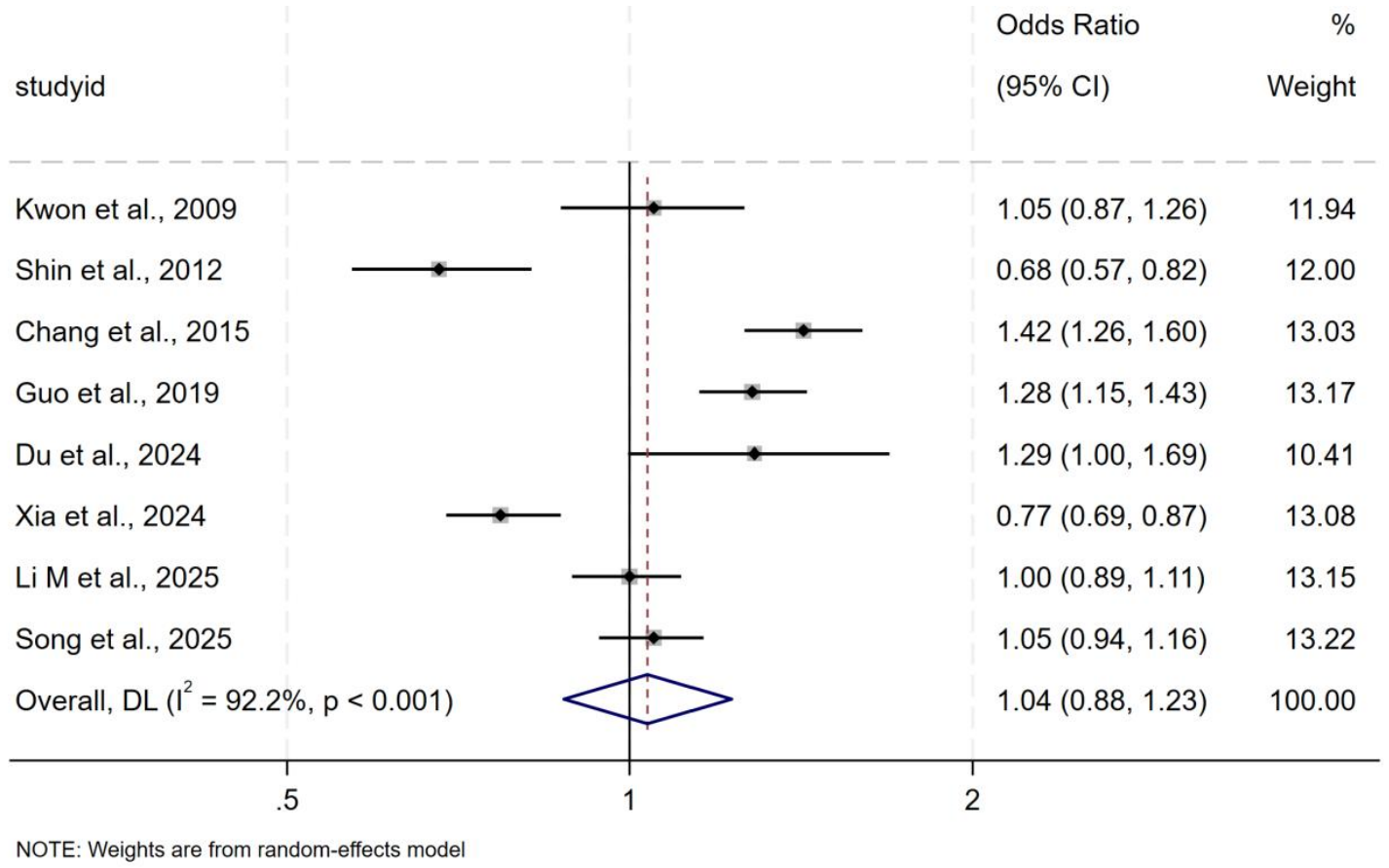

f. Current smoker

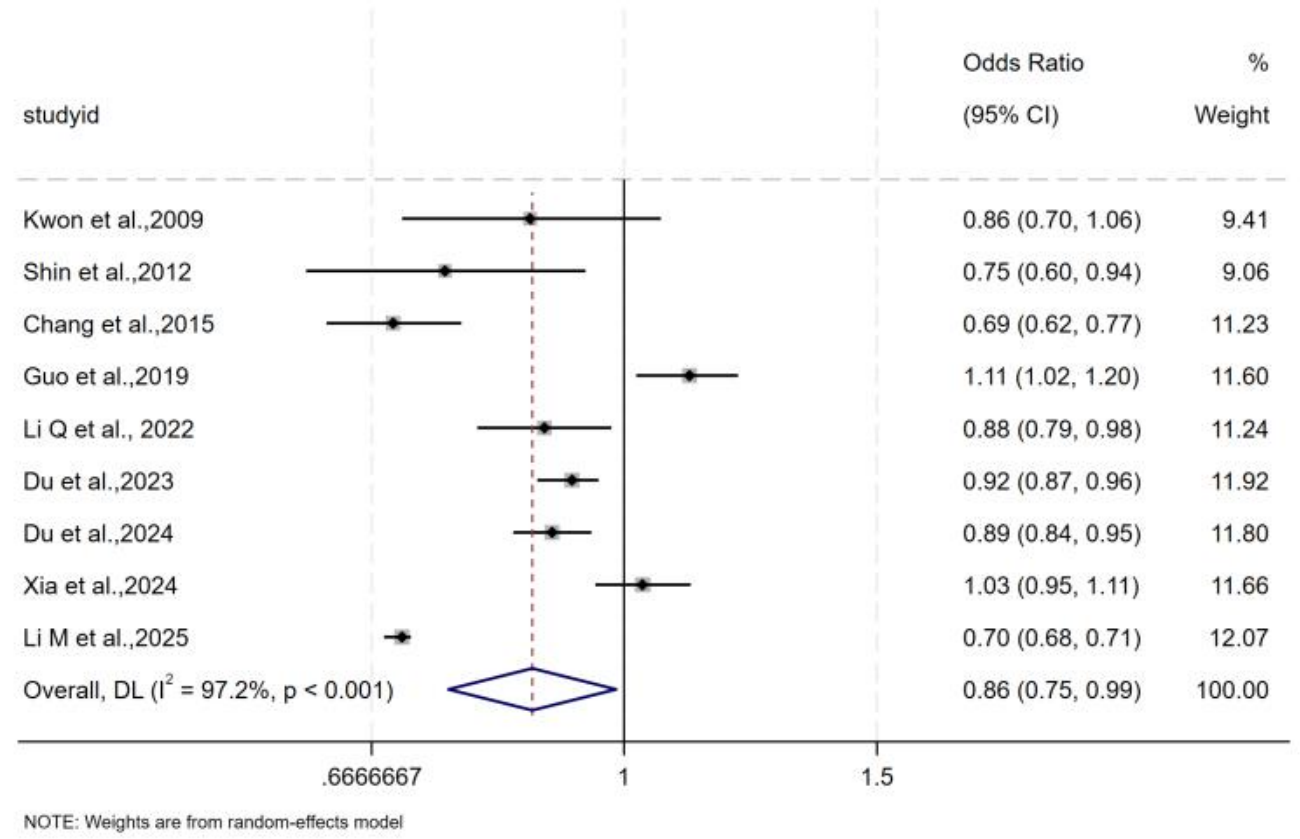

g. Ex-smoker

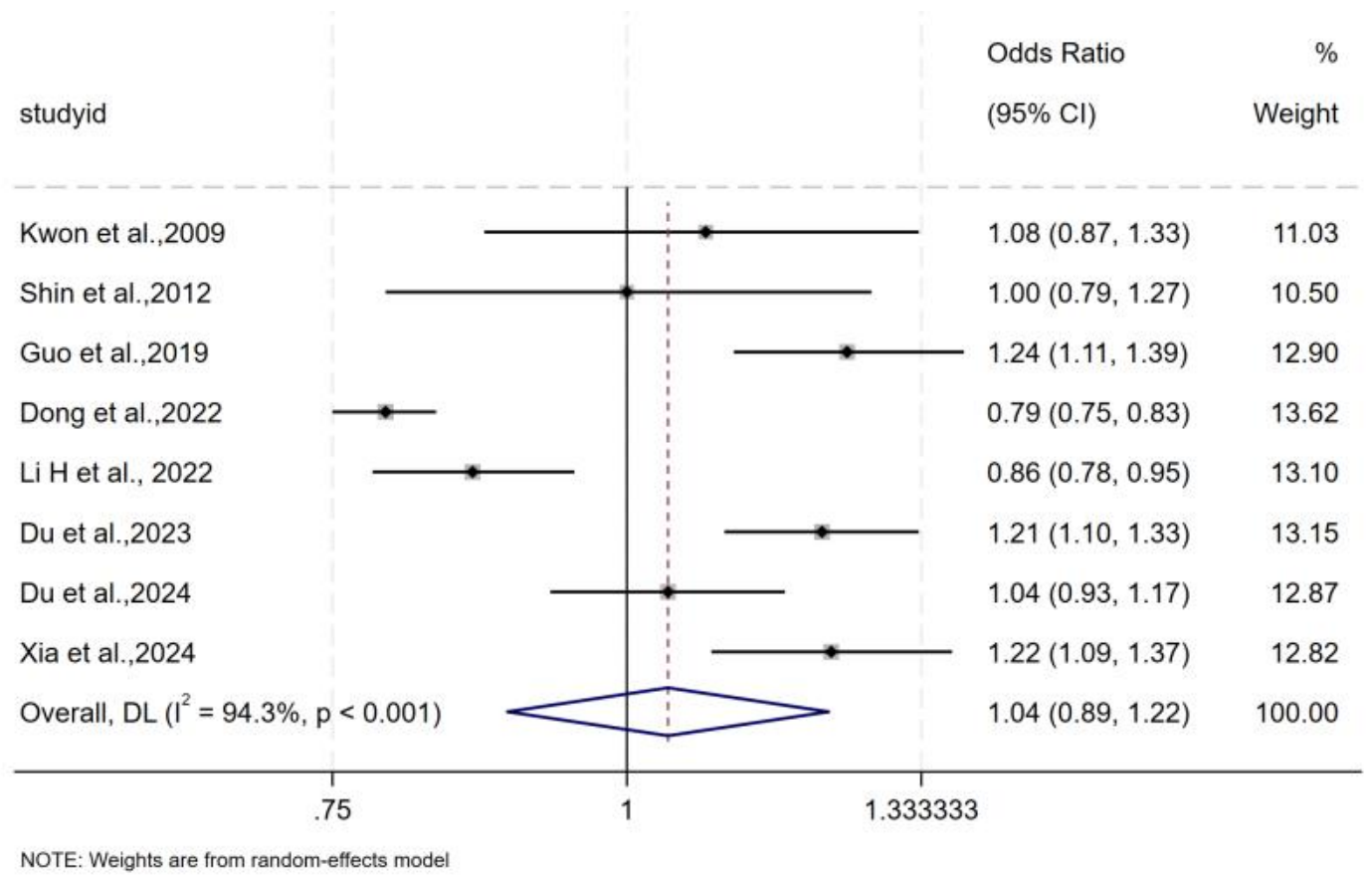

h. Regular physical activity

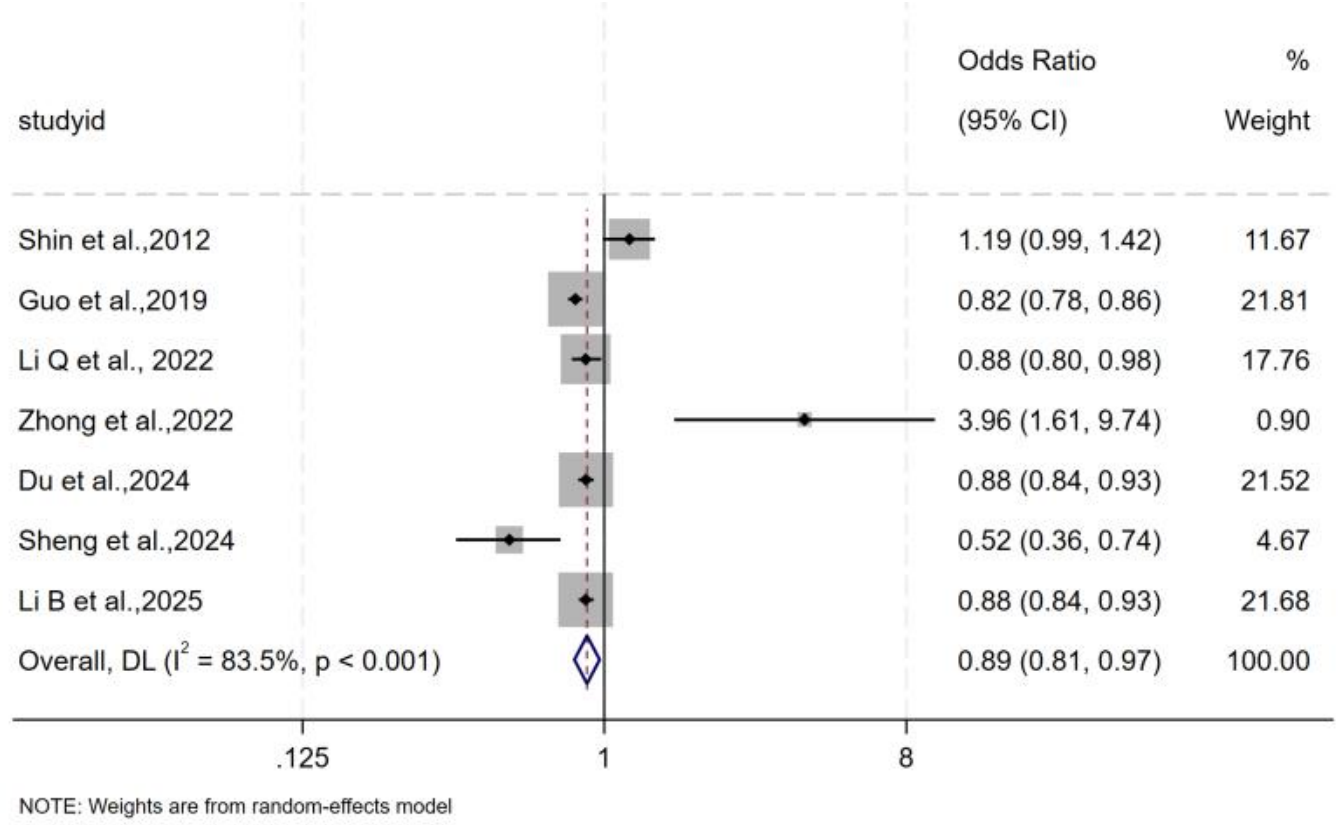

i. Education

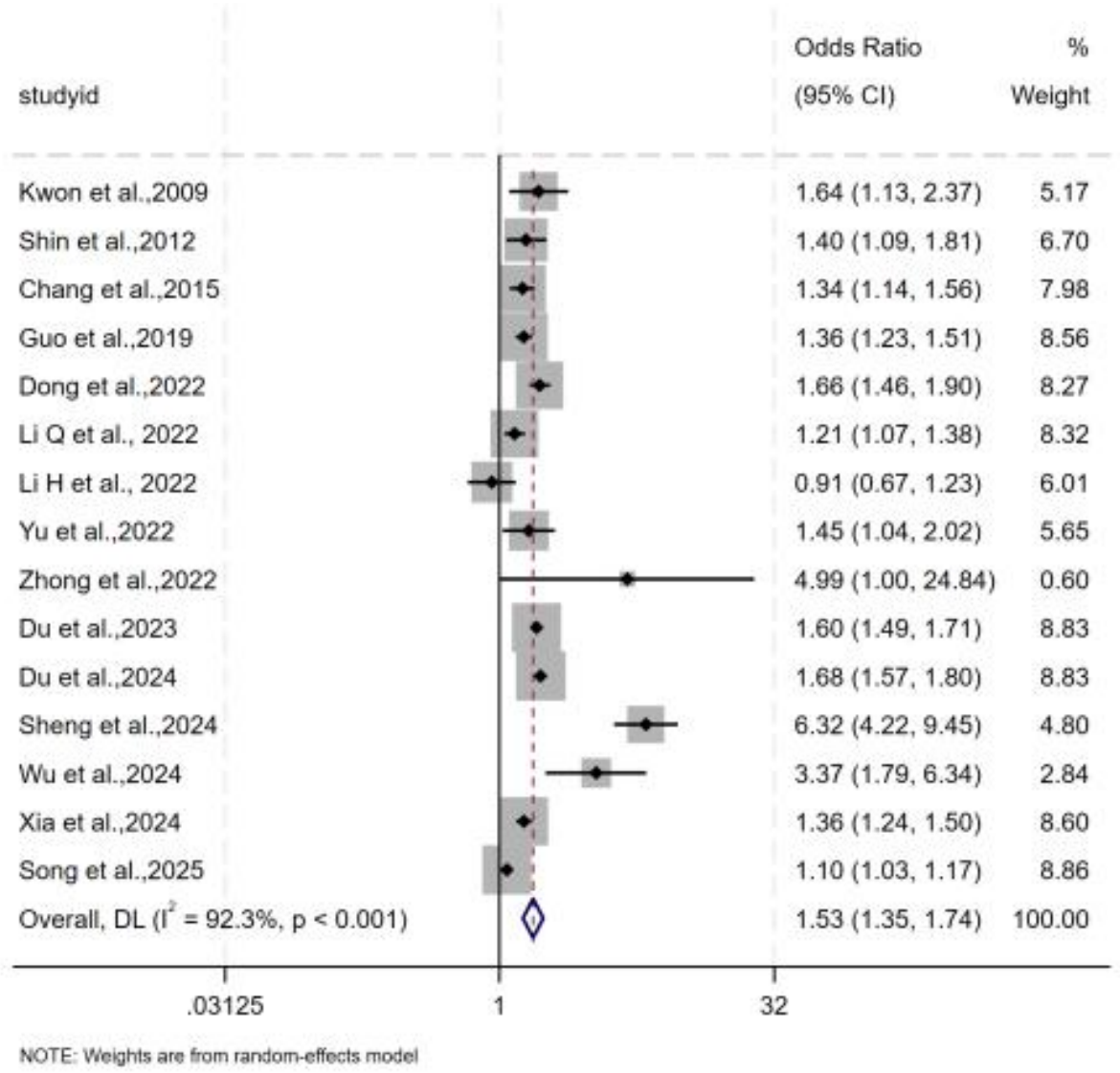

j. Occupational exposure to hazards

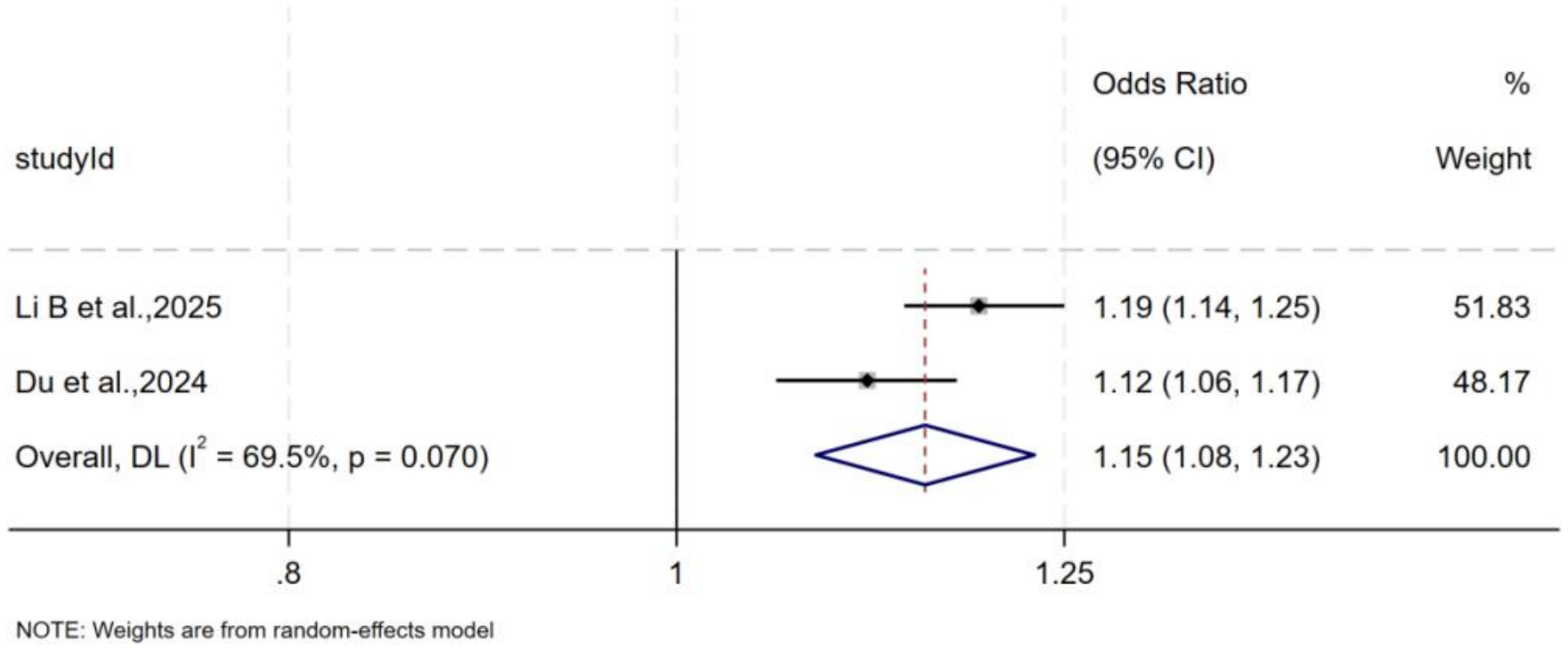

Footnote. Effect estimates were harmonised as higher education versus lower education for endoscopic screening uptake. When multiple education categories were reported within the same study, only the highest-versus-lowest available education comparison was included in the primary meta-analysis to avoid double-counting participants; intermediate categories were retained in the extraction table but not pooled. When stratified estimates were reported for mutually exclusive residence subgroups, subgroup-specific estimates were first combined within the study using a fixed-effect inverse-variance approach. When an original study reported non-uptake rather than uptake, estimates were re-oriented so that all pooled effects referred to endoscopic screening uptake.For Li H et al., 2022, rural and urban estimates were reported separately. Because these strata were mutually exclusive, they were first combined within the study using a fixed-effect inverse-variance approach on the log OR scale and then entered as a single study-level estimate in the primary meta-analysis.

k. Drinking untreated water sources (shallow water)

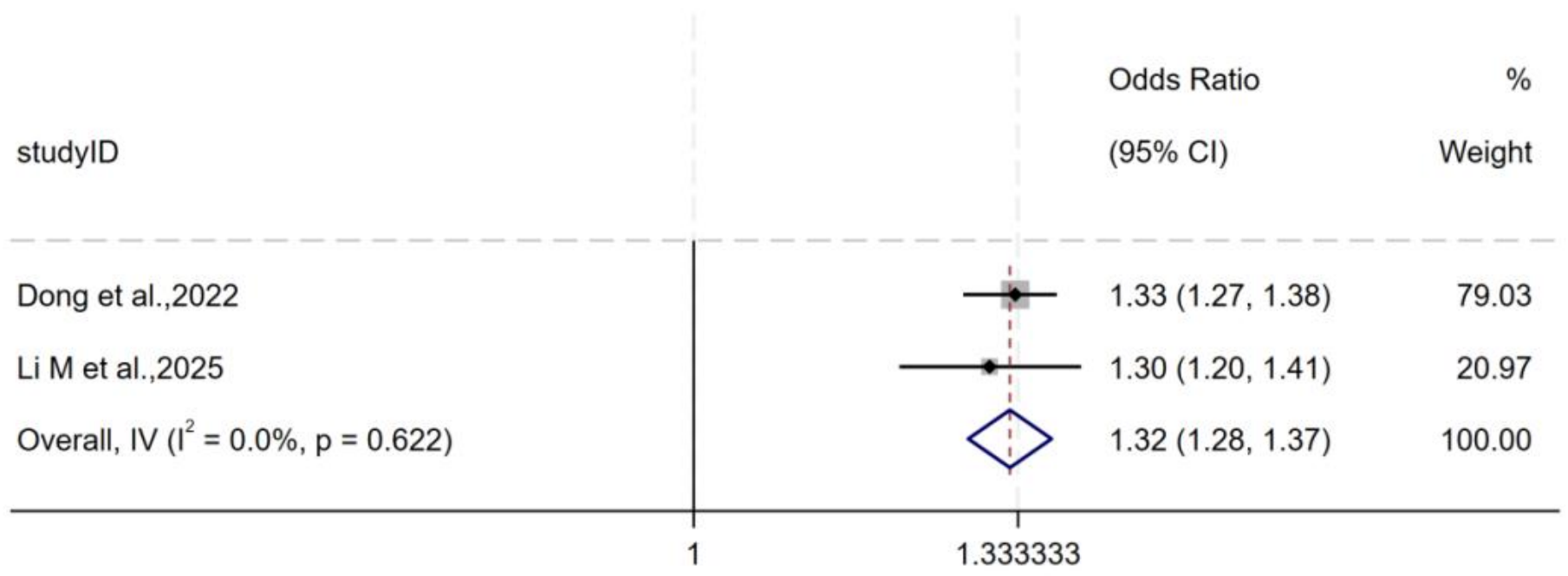

l. Drinking untreated water sources (deep water)

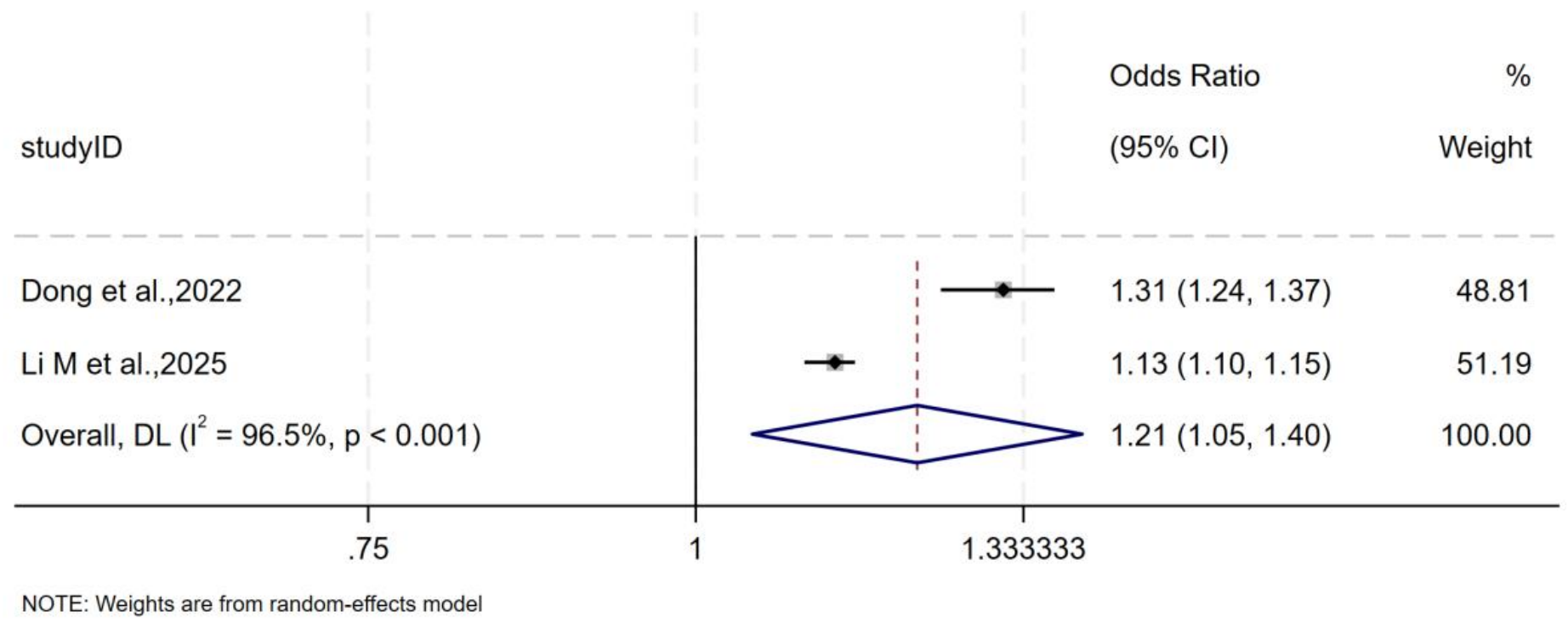

Figure S4. Funnel plot and egger's test of effect sizes against standard errors to assess publication bias

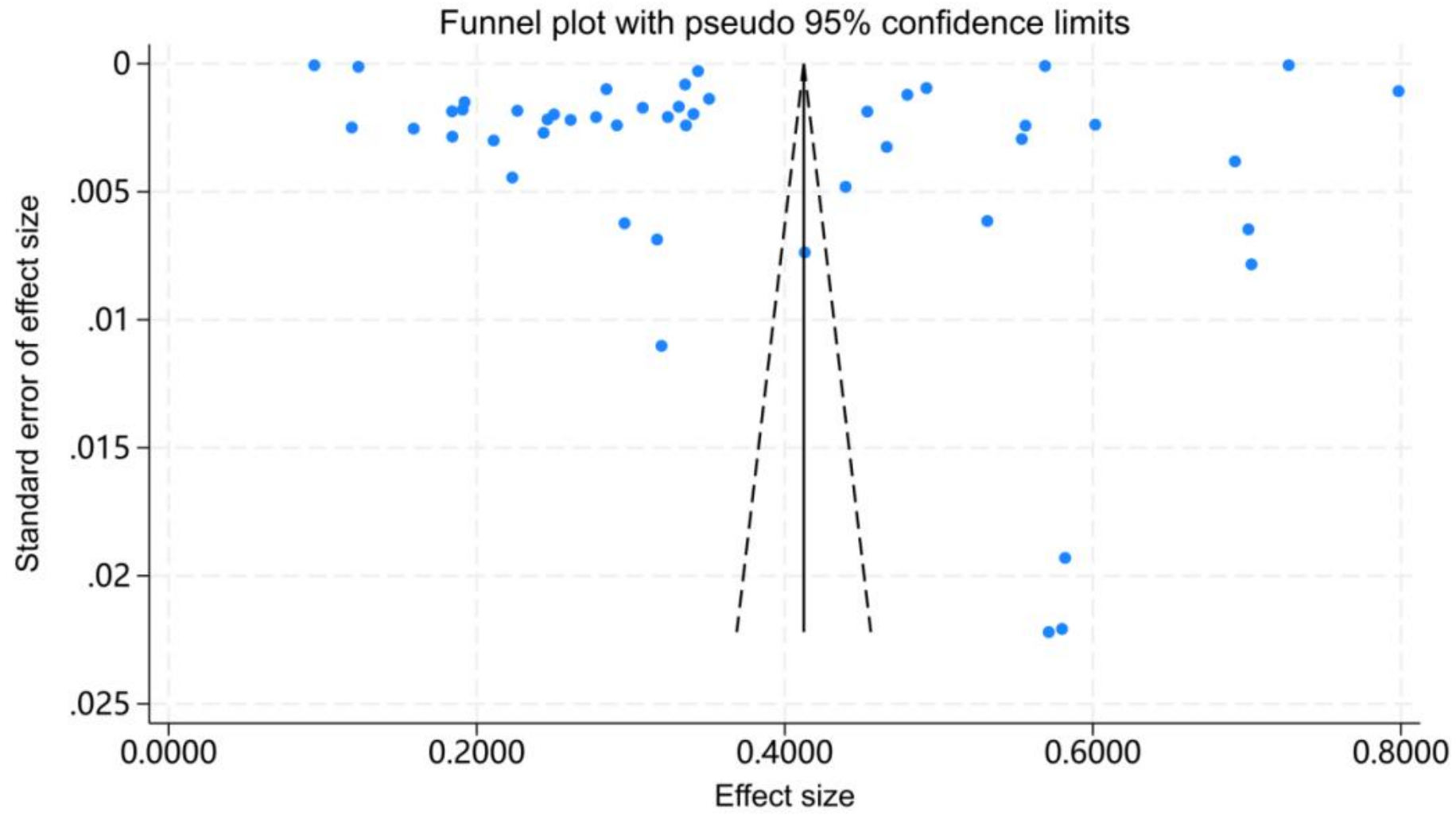

Egger's test for small-study effects:
Regress standard normal deviate of intervention
effect estimate against its standard error

.

Number of studies = **48** Root MSE = **1169**

Std_Eff	Coefficient	Std. err.	t	P>|t|	[95% conf. interval]	
slope	**.4165564**	**.0462287**	**9.01**	**0.000**	**.3235029**	**.5096099**
bias	**-42.76295**	**183.3035**	**-0.23**	**0.817**	**-411.7337**	**326.2078**

Test of H0: no small-study effects P = **0.817**

. metafunnel _ES _seES

Note: default data input format (theta, se_theta) assumed.
